# Exact solutions via Prabhakar fractional approach to investigate heat transfer and flow features of hybrid nanofluid subject to shape and slip effects

**DOI:** 10.1038/s41598-023-34259-9

**Published:** 2023-05-14

**Authors:** Asifa, Talha Anwar, Poom Kumam, Musawa Yahya Almusawa, Showkat Ahmad Lone, Panawan Suttiarporn

**Affiliations:** 1 Department of Mathematics, Faculty of Science, King Mongkut’s University of Technology Thonburi, 126 Pracha-Uthit Road, Bang Mod, Thung Khru, Bangkok 10140 Thailand; 2 Center of Excellence in Theoretical and Computational Science (TaCS-CoE), Science Laboratory Building, Faculty of Science, King Mongkut’s University of Technology Thonburi (KMUTT), 126 Pracha-Uthit Road, Bang Mod, Thung Khru, Bangkok 10140 Thailand; 3 Department of Medical Research, China Medical University Hospital, China Medical University, Taichung, 40402 Taiwan; 4 Department of Mathematics, Faculty of Science, Jazan University, Jazan, 45142 Saudi Arabia; 5 Department of Basic Sciences, College of Science and Theoretical Studies, Saudi Electronic University, Jeddah Campus, Riyadh, 11673 Saudi Arabia; 6 Faculty of Science, Energy and Environment, King Mongkut’s University of Technology North Bangkok, Rayong Campus, Rayong, 21120 Thailand

**Keywords:** Fluid dynamics, Mechanical engineering, Applied mathematics

## Abstract

The core devotion of this study is to develop a generalized model by means of a recently proposed fractional technique in order to anticipate the enhancement in the thermal efficiency of engine oil because of the dispersion of graphene and magnesia nanoparticles. In addition to investigating the synergistic attributes of the foregoing particles, this work evaluates shape impacts for column, brick, tetrahedron, blade, and lamina-like shapes. In the primary model, the flow equation is coupled with concentration and energy functions. This classical system is transmuted into a fractional environment by generalizing mathematical expressions of thermal and diffusion fluxes by virtue of the Prabhakar fractional operator. In this study, ramped flow and temperature slip conditions are simultaneously applied for the first time to examine the behavior of a hybrid nanofluid. The mathematical analysis of this problem involves the incorporation of dimension-independent parameters into the model and the execution of the Laplace transform for the consequent equations. By doing so, exact solutions are derived in the form of Mittag–Leffler functions. Multiple illustrations are developed by dint of exact solutions to chew over all aspects of temperature variations and flow dynamics. For the preparation of these illustrations, the details of parametric ranges are as follows: $0.00 \le \varUpsilon \le 0.04$, $2.0 \le Gr_1 \le 8.0$, $0.5 \le Sc \le 2.0$, $0.1 \le \uptau \le 4.0$, $0.1 \le d \le 0.6$, $0.2 \le \lambda _1 \le 1.5$, and $1.0 \le Gr_2 \le 7.0$. The contribution of differently shaped nanoparticles, volume proportions, and fractional parameters in boosting the heat-transferring attributes of engine oil is also anticipated. In this regard, results for Nusselt number are provided in tabular form. Additionally, a brief analysis of shear stress is carried out for fractional parameters and various combinations of magnesia, graphene, and engine oil. This investigation anticipates that engine oil’s hybridization with magnesia and graphene would result in a 33% increase in its thermal performance, which evidently improves its industrial significance. The enhancement in Schmidt number yields an improvement in the mass transfer rate. An increment in collective volume fraction leads to raising the profile of the thermal field. However, the velocity indicates a decreasing behavior. Nusselt number reaches its highest value ($Nu=8.1363$) for the lamina shape of considered particles. When the intensity of the buoyancy force augments, it causes the velocity to increase.

## Introduction

The specific technological objective of accurately controlling molecules and atoms by employing various tools and techniques to fabricate different macro-scale objects is recognized as nanotechnology. In the contemporary era of progress, where materials and machines are getting smaller every day and accumulating more characteristics and functions, nanotechnology is expanding expeditiously. It offers extensive scientific evolution and facilitates the development and functioning of multiple advanced gadgets and tools in many industries. For example, pharmaceutical industry, oil refineries, nanoelectronics, automobile manufacturing, energy sector, and many others. The most intriguing aspects of nanotechnology for scientists include economic advantages, time and resource efficiency, and amelioration of objects’ features. Researchers from a variety of disciplines, for instance, biomaterials engineering, nanomedicine, organic chemistry, surface science, and energy production discussed the advantages and applications of nanotechnology^1,2^. One of the core constituent parts of nanotechnology is nanofluid, which is predominantly employed to manage heat transfer complications adequately. In this day and age, the acquisition of sufficient temperature control for ultrasensitive equipment throughout numerous industrial operations like thermal insulation, nuclear plants, fiber coating, heat exchangers, and reactor fluidization is the paramount challenge. The regular fluids taking part in these activities lack the necessary features for the disposal of surplus heat. Therefore, experts have devised a number of methodologies to boost the thermal adequacy of these regular fluids. The emergence of nanofluids, which not only serve the cause of escalating heat-transporting potentials but also enhance anti-wear, lubrication, and corrosion prevention characteristics of usual fluids, is credited with a paradigm shift in this domain.

A fluid developed through the immersion of nano-sized particles in regular fluids like oils, water, drilling muds, and lubricants is termed a nanofluid. These particles have diameters below 100 nanometers, and they might be made of carbon nanotubes, oxides (CO$_2$, ZnO, MgO), metals (zinc, silver, iron), or non-metals (silica, graphene). Because of the origination of nanofluids, a plethora of new disciplines, such as molecular engineering, nanophotonics, and materials science, have emerged in several branches of engineering and technology. The immersion of solid particles in regular fluids yields a number of positive results, for instance, the ensuing nanofluids possess effective tribological properties, enhanced lubricating potentials, and they perform better in terms of thermal management. These momentous advantages support nanofluids as a viable replacement for regular fluids for diverse operations and equipment such as microelectronics, household refrigeration systems, optical sensors, combustion operations, and heat exchangers. In addition to this, a valuable approach to boosting the productivity of several industrial and commercial tools and systems, for instance, electronic devices, transformers, vehicle radiators, energy accumulators, solar collectors, and power stations, is to utilize nanofluids instead of regular fluids.

During the current decade, innumerable analytic and experimental investigations have been carried out to evaluate diverse aspects of nanofluids. Subramanian et al.^3^ examined the pressure drop and heat-carrying performance of TiO$_2$–H$_2$O nanofluid in turbulent, transitional, and laminar flow domains. They observed an augmentation of 25% in the thermal behavior of water along with a slightly higher pressure drop because of TiO$_2$ nanoparticles. Hussain et al.^4,5^ compared the behavior of multiple engine oil based nanofluids in a rotating frame and also discussed the impacts of a partial slip condition for flow over a stretching sheet. In one study, they reported that zinc oxide nanoparticles are more efficacious than titanium oxide particles in terms of improving the performance of engine oil. In the latter study, they concluded that the heat transfer rate of engine oil and copper based nanofluid is higher than that of alumina based nanofluid. Prasannakumara^6^ employed a numerical method to conduct a comparative thermal analysis of viscous and Maxwell nanofluids. He observed that the thermal efficacy of viscous nanofluid is significantly higher than that of Maxwell nanofluid. The variation in thermal, mass, and flow conduct of Williamson nanofluid due to convective conditions and Lorentz force was studied by Srinivasulua and Goud^7^. They carried out this analysis for a stretching sheet and discussed that strong magnetic effects result in the reduction of both flow and thermal functions. Usafzai et al.^8^ derived multiple solutions to investigate flow and thermal fields of nanofluids, considering temperature jump and slip flow effects. They remarked that the flow gets retarded for the dominant slip influence, whereas the thermal distribution exhibits an opposite result for an increased fraction of nanoparticles. Jamshed et al.^9^ compared alumina and copper based second grade nanofluids subject to several additional impacts, such as heat radiation, viscous dissipation, porosity of medium, and heat source. They analyzed that copper based nanofluid is relatively more efficient regarding heat-transportation purposes. Urmi et al.^10^ provided an extensive review about preparation, stability, challenges, and applications of nanofluids. Some important results on different features of nanofluids are presented in^11–14^.

Despite the fact that crucial needs of industrial processes can be adequately fulfilled through nanofluids due to their advanced attributes, scientists pursued their research in order to prepare a more useful fluid. This pursuit led to the production of a new fluid, termed a hybrid nanofluid. It is engineered via the immersion of two different nanoparticles in a regular fluid, and the process is often called hybridization. Hybridization results in a trade-off between the drawbacks and advantages of individual inclusion of nanoparticles, which influences the material features of regular fluids in a favorable manner. Amelioration in heat transportation rates, diminution in friction effects, and advancements in thermal traits are some of the key benefits of hybridization. Hybrid nanofluids are utilized in a variety of fields and processes, for instance, machine coolants and lubricants, ventilation, hybrid engines, automotive industry, cooling of generators and transformers, drilling and grinding, refrigeration, energy storage instruments, heat pipes, and cooling of nuclear systems. Numerous experimental and mathematical research works have been organized to explain the applications, performances, and advantages of various hybrid nanofluids. Ali et al.^15^ explored the control of Hall current and slippage condition on the peristaltic motion of a copper and titanium dioxide based hybrid nanofluid in an unsymmetrical channel. A hybrid nanofluid consisting of graphene and ferrous nanoparticles was comprehensively evaluated by Acharya and Mabood^16^. They claimed that the dispersion of these particles in water produces a 74.25% enhancement in Nusselt number. The impacts of multiple physical phenomena, such as thermal absorption, magnetic field, velocity slippage, chemical reaction, and ramped thermal function, on Casson hybrid nanofluid flow in a rotating frame were examined by Krishna et al.^17^. Kanti et al.^18^ applied an experimental approach to particularly analyze hybrid nanofluids composed of graphene oxide. They chewed over specific modifications in material features, stability, and thermal applications of these fluids. Chu et al.^19^ anticipated the thermal conductivity of gold-silver based hybrid nanofluid by dint of two different mathematical models and compared flow and heat transmitting performances for an infinite vertical channel. Shah and Ali^20^ discussed the problems and limitations of using hybrid nanofluids in solar systems. Eid and Nafe^21^ provided several graphs to dissect the consequences of heat injection and thermal properties variation. In this work, the examined hybrid nanofluid contains ethylene glycol, copper, and magnetite. An all-inclusive review on heat transfer, entropy production, and convective flows of hybrid/nanofluids was communicated by Al-Chlaihawi et al.^22^. Further recent works on hybrid nanofluids can be accessed from^23–26^.

Generally, the structures and loading fractions of nanoparticles, their types and shapes, and the intrinsic features of the involved fluids are some of the pivotal elements that affect a hybrid nanofluid’s functionality. Realizing the importance of these contributing factors, a crucial concern that emerges here is which shape will be more suitable to achieve the maximum boost in thermal attributes. An exhaustive survey of the literature conveys that few studies evaluate hybrid nanofluids subject to shape influences, which highlights a dearth of inspections on this topic. Furthermore, it is essential to comprehend that theoretical examinations that do not take shape factors into consideration are less useful in terms of practical applications. Ghadikolaei et al.^27^ carried out a numerical analysis to evaluate the role of brick, platelet, and cylinder shaped titania and copper nanoparticles in the development of stagnation flow. The dominance of different shapes of titanium dioxide and silver nanoparticles on heat transfer characteristics for flow in a horizontal tube was accessed and elucidated by Benkhedda et al.^28^. Saba et al.^29^ studied the thermal conduct of Al$_2$O$_3$–Cu/water hybrid nanofluid in an unsymmetrical channel subject to walls’ dilation/contraction and multiple shape effects. They worked with platelet, brick, and cylinder-like shapes. Alarabi et al.^30^ further examined the aforementioned hybrid nanofluid for sphere, hexahedron, lamina, tetrahedron, and column shapes. They utilized a single-phase model and considered a cylindrical geometry for this investigation. Ramzan et al.^31^ compared graphene-silver and graphene-copper oxide hybrid nanofluids to analyze thermophysical variations occurring because of cylindrical shaped graphene, palatelet-like silver, and spherically shaped copper oxide nanoparticles.

Magnesia and graphene nanoparticles have certain significant features that make them useful for a variety of engineering and industrial applications. For instance, graphene possesses a large surface area to volume ratio, which makes it ideal for use in applications where surface area is important, such as catalysis and heat transfer. Graphene nanoparticles are highly conductive, meaning they can easily conduct heat and electricity. Due to this feature, they are highly effective for electronics and energy storage applications. These particles are incredibly strong and rigid therefore, their use for reinforcement in coatings and composites provides desirable outcomes. Furthermore, they can be utilized in sensors, drug delivery, water filtration, and tissue engineering. On the other hand, magnesia is thermally stable at extremely high temperatures because it offers significant resistance to current and has a high potential for heat conduction. These particles are also biocompatible therefore, they are effective for certain biomedical applications like imaging and cancer therapy. Also, the production of specific optical materials that are used to treat indigestion and heartburn involves magnesia. Since magnesia offers resistance to moisture and fire, it is one of the fundamental components of construction materials.

The concept of adopting fractional methods to modify regular models gave rise to a new field called fractional calculus. Due to its extensive implementations in diverse real-life conditions, it is a discipline that is now undergoing rapid evolution. In recent times, multiple experts from various scientific fields have reported that results procured by the virtue of fractional approaches are more reliable, and the execution of fractional operators for modeling purposes ensures the specificity and accuracy of outcomes. In addition, they offer a more precise interpretation of the under-observation process. Besides, a fitting adjustment of fractional parameters leads to a good accordance between theoretically derived solutions and experimentally established findings. These additional benefits have motivated a number of researchers to examine physical problems in fractional environments and perform comparative investigations. The usages of fractional models can be found in a variety of areas such as dynamical systems, control theory, disease and population modeling, electromagnetics, economics, fluid mechanics, mathematical biology, and so forth. Regarding fluid mechanics, memory and self-similar effects of fractional derivatives are significantly crucial to fully comprehend the rheological features, thermal performances, and viscoelastic behaviors of fluids. So far, a variety of fractional operators composed of various mathematical formulations have been presented. Each of them has unique limitations and benefits. In this list, Caputo and Riemann–Liouville are the most frequently utilized operators, and their formulations involve a power-law kernel^32^. The other well-known operators are Prabhakar, Atangana-Baleanu, Hilfer, Caputo-Fabrizio, Hadamard, and Grüünwald-Letnikov fractional operators^33–35^. As compared to standard methods, modern-day researchers are preferring fractional modeling techniques in order to provide more authentic descriptions of physical mechanisms based on generalized solutions. Several fractional operators have been utilized until now to chew over the complexities of diverse natural phenomena. Fallahgoul et al.^36^ analyzed the impacts of vital characteristics of fractional processes, for instance, self-similarity, path-dependency, and long-range memory, on financial theory, economics, and financial models. Sinan et al.^37^ used the Atangana-Baleanu operator to establish a model for an in-depth investigation of the malaria disease. They discussed the effectiveness of precautionary measures and medication for the reduction of the disease’s spread. Asjad et al.^38^ evaluated the control of generalized boundary conditions on heat-conducting properties of nanofluids by means of a fractional system. Raza et al.^39^ explained the thermal aspects of water and kerosene oil based nanofluids with the aid of semi-analytic fractional solutions. Ikram et al.^40^ established multiple fractional models to explore the heat-transmitting efficiency of hybrid nanofluids during channel flows. Some of the latest studies in which thermal and flow distributions are examined via fractional operators can be viewed in^41–44^.

A meticulous analysis of the literature reveals that there are not enough studies on such hybrid nanofluids, which contain oils as host fluids. This research gap further expands if the utilization of fractional operators and the derivation of exact solutions are simultaneously taken into consideration. In addition, it is noted that shape aspects of nanoparticles have not been given adequate importance because most of the reported investigations communicate results for spherical structures. This work is an attempt to address all these concerns. The principal feature of this analysis is to investigate the consequences of hybridizing magnesia and graphene nanoparticles with engine oil. The shapes’ influences are given significant attention. In this regard, it is assumed that the observed particles have blade, column, lamina, brick, and tetrahedron-like shapes. This research work also aims to explain the flow dynamics and thermal behavior of the consequent hybrid nanofluid in terms of a fractional model. To serve this purpose, generalized relations for thermal and diffusion fluxes are established with the aid of the Prabhakar fractional operator. The inclusion of dimension-independent quantities in the principal system lays the foundation for the implementation of the fractional operator. In this work, the behavior of hybrid nanofluid for simultaneous application of ramped flow and thermal slip conditions is examined for the first time. To solve the ensuing fractional system, the Laplace transformation is executed, and exact solutions are produced in the form of multi-parametric Mittag–Leffler functions. Various tables and graphical illustrations are presented to effectively evaluate flow patterns, thermal profiles, shape impacts, contribution of influential parameters, concentration field, and heat transfer performance. The illustrations are compared for slip and no-slip cases and for lower and higher time values to emphasize the importance of slip conditions, transient effects, and the ramped velocity condition. Some modifications in fractional parameters are performed to retrieve thermal and flow functions for the classical case, and their graphical comparison is carried out with those acquired via the fractional model.

## Statement and model formulation

**Figure 1:**
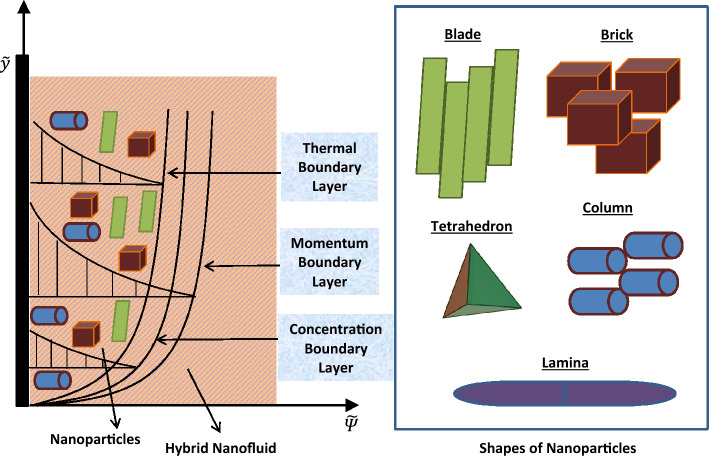
Geometrical setting of this study.

In this work, the hybridization of multi-shaped magnesia and graphene nanoparticles with engine oil is examined to discuss the significant modifications in thermal and flow characteristics of engine oil. The geometrical arrangement for this analysis involves an infinite upright wall that serves as the solid-fluid interface. Initially, the temperature of hybrid nanofluid is $\Theta _\infty$ with a uniform concentration ${\mathcal {C}}_\infty$, and the system demonstrates no motion. Later on, ramped movement of the bounding wall and a temperature variation due to slip effects disturb the system. In the mathematical sense, a piece-wise function describes the ramped movement in such a way that the velocity is a time-dependent function ($U_0({\widetilde{\uptau }}/{\uptau _0})$) for a particular duration $({\widetilde{\uptau }} \le \uptau _0)$. After that (for ${\widetilde{\uptau }} > \uptau _0$), the velocity has a constant value ($U_0$). Meanwhile, the concentration changes from ${\mathcal {C}}_\infty$ to ${\mathcal {C}}_w$. Far from the wall, the flow function associated with hybrid nanofluid attains a zero value, and thermal and concentration functions again achieve ambient values ($\Theta _\infty$ and ${\mathcal {C}}_\infty$). Figure 1 provides the geometrical setting of this study. The mathematical model is developed considering the following assumptions

- The flow, concentration, and thermal functions only contain one axial component (${\widetilde{\varPsi }}$) due to the infinite length of the bounding wall.
- The flow is one-dimensional and unidirectional.
- Engine oil is in thermal equilibrium with magnesia and graphene nanoparticles.
- Nanoparticles are assumed to have brick, column, lamina, blade, and tetrahedron shapes.
- The viscosity dissipation does not disturb the heat transfer process.
- The buoyancy effects are addressed using the Boussinesq approximation^45^.

Taking the aforestated assumptions and description into consideration, the principal equations for this problem are derived as^46^

1 $$\begin{aligned} \breve{\rho }_{hnf}\left( 1+{\widetilde{\lambda }}\frac{\partial }{\partial {\widetilde{\uptau }}} \right) \frac{\partial {{\widetilde{U}}} \big ({\widetilde{\varPsi }},{\widetilde{\uptau }} \big )}{\partial {\widetilde{\uptau }}}&=\left( 1+{\widetilde{\lambda }} \frac{\partial }{\partial {\widetilde{\uptau }}} \right) \big (\breve{\rho }\breve{\beta }_\Theta \big )_{hnf}\big ({\widetilde{\Theta }}\big ({\widetilde{\varPsi }},{\widetilde{\uptau }} \big )-\Theta _\infty \big )g \qquad \qquad \qquad \qquad \nonumber \\&\quad +\breve{\mu }_{hnf} \frac{\partial ^2 {\widetilde{U}} \big ({\widetilde{\varPsi }},{\widetilde{\uptau }} \big )}{\partial {{\widetilde{\varPsi }}}^2} +\left( 1+{\widetilde{\lambda }} \frac{\partial }{\partial {\widetilde{\uptau }}}\right) \big (\breve{\rho }\breve{\beta }_{{\mathcal {C}}}\big )_{hnf}\big (\mathcal {{\widetilde{C}}}\big ({\widetilde{\varPsi }},{\widetilde{\uptau }} \big )-{\mathcal {C}}_\infty \big )g, \end{aligned}$$

2 $$\begin{aligned} \frac{\partial {{\widetilde{\Theta }}}\big ({\widetilde{\varPsi }},{\widetilde{\uptau }} \big )}{\partial {\widetilde{\uptau }}}&=-\frac{1}{\big (\breve{\rho } \breve{{\textsf{C}}}_p\big )_{hnf} }\frac{\partial {\widetilde{q}}\big ({\widetilde{\varPsi }},{\widetilde{\uptau }} \big )}{\partial {\widetilde{\varPsi }}}, \qquad \qquad \qquad \qquad \qquad \quad \end{aligned}$$

3 $$\begin{aligned} \frac{\partial {\mathcal { {\widetilde{C}}}}\big ({\widetilde{\varPsi }},{\widetilde{\uptau }} \big )}{\partial {\widetilde{\uptau }}}&=-\frac{\partial {\mathcal { {\widetilde{J}}}}\big ({\widetilde{\varPsi }},{\widetilde{\uptau }} \big )}{\partial {\widetilde{\varPsi }}}. \qquad \qquad \qquad \qquad \qquad \qquad \end{aligned}$$

Fourier’s law for thermal flux and Fick’s law for diffusion equation are respectively provided as

4 $$\begin{aligned} \left( \frac{1}{\breve{{\mathcal {K}}}_{hnf}} \right) {\widetilde{q}}\big ({\widetilde{\varPsi }},{\widetilde{\uptau }} \big )&=-\frac{\partial {\widetilde{\Theta }} \big ({\widetilde{\varPsi }},{\widetilde{\uptau }} \big )}{\partial {\widetilde{\varPsi }}}, \end{aligned}$$

5 $$\begin{aligned} \left( \frac{1}{\breve{{\mathcal {D}}}_{hnf}} \right) \widetilde{{\mathcal {J}}}\big ({\widetilde{\varPsi }},{\widetilde{\uptau }} \big )&=-\frac{\partial \widetilde{{\mathcal {C}}} \big ({\widetilde{\varPsi }},{\widetilde{\uptau }} \big )}{\partial {\widetilde{\varPsi }}}. \end{aligned}$$

The following conditions are associated to the above-mentioned system of equations

6 $$\begin{aligned} \text {For} \quad {\widetilde{\uptau }}&= 0: \quad {\widetilde{U}}({\widetilde{\varPsi }},{\widetilde{\uptau }})=0, \quad {\widetilde{\Theta }}({\widetilde{\varPsi }},{\widetilde{\uptau }})=\Theta _\infty , \quad {\mathcal {{\widetilde{C}}}}({\widetilde{\varPsi }},{\widetilde{\uptau }})={\mathcal {C}}_\infty , \quad \qquad \quad \end{aligned}$$

7 $$\begin{aligned} \text {For} \quad {\widetilde{\varPsi }}&= 0: \quad {\widetilde{U}}({\widetilde{\varPsi }},{\widetilde{\uptau }})=\left\{ \begin{array}{ll} U_0\frac{{\widetilde{\uptau }}}{\uptau _0} \quad 0 < {\widetilde{\uptau }} \le \uptau _0 \\ U_0 \qquad \qquad {\widetilde{\uptau }}>\uptau _0 \end{array}\right. , \quad \mathcal {{\widetilde{C}}}({\widetilde{\varPsi }},{\widetilde{\uptau }})={\mathcal {C}}_w, \nonumber \\&\quad {\widetilde{\Theta }}({\widetilde{\varPsi }},{\widetilde{\uptau }})-d^* \frac{\partial {\widetilde{\Theta }}({\widetilde{\varPsi }},{\widetilde{\uptau }})}{\partial {\widetilde{\varPsi }}}=\Theta _\infty +h({\widetilde{\uptau }})(\Theta _w-\Theta _\infty ), \quad \end{aligned}$$

8 $$\begin{aligned} \text {For} \quad {\widetilde{\varPsi }}&\rightarrow \infty : \quad {\widetilde{U}}({\widetilde{\varPsi }},{\widetilde{\uptau }}) \rightarrow 0, \quad {\widetilde{\Theta }}({\widetilde{\varPsi }},{\widetilde{\uptau }}) \rightarrow \Theta _\infty , \quad \mathcal {{\widetilde{C}}}({\widetilde{\varPsi }},{\widetilde{\uptau }}) \rightarrow {\mathcal {C}}_\infty . \qquad \quad \end{aligned}$$

## Mathematical relations for thermal and physical properties

The major factors that make a hybrid nanofluid preferable to a standard industrial fluid are its enhanced physical and thermal characteristics. These features of the involved nanoparticles significantly influence the development of flow and have a substantial impact regarding the thermal usability of the consequent hybrid nanofluid. However, when it comes to describing all these features mathematically, there is no universal model that can address them simultaneously. However, a number of researchers tried to study different aspects of these features through experiments. As a result, several mathematical relations have been established to explain the thermo-physical attributes of nanoparticles. Later on, to adequately characterize these properties of hybrid nanofluids, these models were effectively adapted. This section primarily focuses on outlining a fundamental mathematical relationship for each thermo-physical feature and its alteration in the case of hybrid nanoparticles.

### Viscosity

Various factors, such as density, drag effect, and viscous forces, contribute to the formation of flow patterns. Additionally, it is crucial to anticipate the ability of a nanofluid to oppose the deformation. In this regard, Brinkman proposed a model in 1952^47^ that received significant attention later, and it is the most frequently applied model in recent times. It anticipates the viscosity as

9 $$\begin{aligned} \breve{\mu }_{{nf}}={\breve{\mu }_{{hf}}} \frac{1}{ \big (1-\varUpsilon _{\text {NP}} \big )^{2.5} }. \end{aligned}$$

The expression for the viscosity of the considered hybrid nanofluid is defined as

10 $$\begin{aligned} \breve{\mu }_{hnf}=\breve{\mu }_{\text {Eo}}\frac{\Big (1-\varUpsilon _{\text {MgO}} \Big )^{-\frac{25}{10}}}{ \Big (1-\varUpsilon _{\text {Gra}} \Big )^{\frac{25}{10}}}. \end{aligned}$$

### Specific heat capacity and thermal and concentration expansion coefficients

The following equations are used to explain specific heat capacity and thermal and concentration expansion coefficients

11 $$\begin{aligned} \big (\breve{\rho } \breve{{\textsf{C}}}_p \big )_{{nf}}&=\big (\breve{\rho } \breve{{\textsf{C}}}_p \big )_{{hf}} \left[ 1+\left\{ \frac{ \big (\breve{\rho } \breve{{\textsf{C}}}_p \big )_{\text {NP}}}{\big (\breve{\rho } \breve{{\textsf{C}}}_p \big )_{{hf}}}-1 \right\} \varUpsilon _{\text {NP}} \right] , \end{aligned}$$

12 $$\begin{aligned} \big (\breve{\rho } \breve{\beta }_\Theta \big )_{{nf}}&=\big (\breve{\rho } \breve{\beta }_\Theta \big )_{{hf}} \left[ 1+\left\{ \frac{ \big (\breve{\rho } \breve{\beta }_\Theta \big )_{\text {NP}}}{\big (\breve{\rho } \breve{\beta }_\Theta \big )_{{hf}}} -1 \right\} \varUpsilon _{\text {NP}} \right] , \end{aligned}$$

13 $$\begin{aligned} \big (\breve{\rho } \breve{\beta }_{{\mathcal {C}}} \big )_{{nf}}&=\big (\breve{\rho } \breve{\beta }_{{\mathcal {C}}} \big )_{{hf}} \left[ 1 +\left\{ \frac{ \big (\breve{\rho } \breve{\beta }_{{\mathcal {C}}} \big )_{\text {NP}}}{\big (\breve{\rho } \breve{\beta }_{{\mathcal {C}}} \big )_{{hf}}}-1 \right\} \varUpsilon _{\text {NP}} \right] . \end{aligned}$$

The altered forms of the above-mentioned relations for hybrid nanofluid are as follows

14 $$\begin{aligned} \big (\breve{\rho } \breve{{\textsf{C}}}_p \big )_{{hnf}}&=\big (1-\varUpsilon _{\text {Gra}} \big )\big (\breve{\rho } \breve{{\textsf{C}}}_p \big )_{\text {Eo}} \left[ 1 +\left\{ \frac{\big (\breve{\rho } \breve{{\textsf{C}}}_p \big )_{\text {MgO}}}{\big (\breve{\rho } \breve{{\textsf{C}}}_p\big )_{\text {Eo}}}-1 \right\} \varUpsilon _{\text {MgO}} \right] +\varUpsilon _\text {Gra}\big (\breve{\rho } \breve{{\textsf{C}}}_p \big )_{\text {Gra}}, \end{aligned}$$

15 $$\begin{aligned} \big (\breve{\rho } \breve{\beta }_\Theta \big )_{{hnf}}&=\big (1-\varUpsilon _{\text {Gra}} \big )\big (\breve{\rho }\breve{\beta }_\Theta \big )_{\text {Eo}} \left[ 1 +\left\{ \frac{ \big (\breve{\rho }\breve{\beta }_\Theta \big )_{\text {MgO}}}{\big (\breve{\rho }\breve{\beta }_\Theta \big )_{\text {Eo}}}-1 \right\} \varUpsilon _{\text {MgO}} \right] +\varUpsilon _\text {Gra}\big (\breve{\rho } \breve{\beta }_\Theta \big )_{\text {Gra}}, \end{aligned}$$

16 $$\begin{aligned} \big (\breve{\rho } \breve{\beta }_{{\mathcal {C}}} \big )_{{hnf}}&=\big (1-\varUpsilon _{\text {Gra}} \big )\big (\breve{\rho }\breve{\beta }_{{\mathcal {C}}} \big )_{\text {Eo}} \left[ 1+\left\{ \frac{ \big (\breve{\rho }\breve{\beta }_{{\mathcal {C}}} \big )_{\text {MgO}}}{\big (\breve{\rho }\breve{\beta }_{{\mathcal {C}}} \big )_{\text {Eo}}}-1 \right\} \varUpsilon _{\text {MgO}} \right] +\varUpsilon _\text {Gra}\big (\breve{\rho } \breve{\beta }_{{\mathcal {C}}} \big )_{\text {Gra}}. \end{aligned}$$

### Density

The expression for an adequate estimation of the density is provided as

17 $$\begin{aligned} \breve{\rho }_{{nf}}=\breve{\rho }_{{hf}} \left[ 1+\left( \frac{ \breve{\rho }_{\text {NP}}}{\breve{\rho }_{hf}}-1 \right) \varUpsilon _{\text {NP}} \right] . \end{aligned}$$

The modification of the above-mentioned expression for magnesia and graphene based hybrid nanofluid is presented as

18 $$\begin{aligned} \breve{\rho }_{hnf}=\big (1-\varUpsilon _{\text {Gra}} \big )\breve{\rho }_{\text {Eo}} \left[ 1 +\left( \frac{ \breve{\rho }_{\text {MgO}}}{\breve{\rho }_{\text {Eo}}}-1 \right) \varUpsilon _{\text {MgO}} \right] +\varUpsilon _\text {Gra}\big (\breve{\rho }_{\text {Gra}} \big ). \end{aligned}$$

### Thermal conductivity

In general, thermal conductivity has a strong impact on the efficiency of a nanofluid. Thus, accurate measurement of thermal conductivity is quite essential. The model introduced by Hamilton and Crosser^48^ is currently the leading model for the quantification of thermal conductivity. This model also counters shape influences effectively therefore, most of the researchers prefer it when objectives include the evaluation of shape effects. This model relates the thermal conductivity of nanofluid, host fluid, and nanoparticles, and the shape factor in the following way

19 $$\begin{aligned} \breve{{\mathcal {K}}}_{{nf}}&=\left\{ \frac{\varUpsilon _{\text {NP}}(s-1)\big (\breve{{\mathcal {K}}}_{\text {NP}}-\breve{{\mathcal {K}}}_{\text {hf}}\big )+\breve{{\mathcal {K}}}_{\text {NP}} +\breve{{\mathcal {K}}}_{{hf}}(s-1)}{\varUpsilon _{\text {NP}}\big (\breve{{\mathcal {K}}}_{{hf}} -\breve{{\mathcal {K}}}_{\text {NP}} \big )+\breve{{\mathcal {K}}}_{\text {NP}}+\breve{{\mathcal {K}}}_{{hf}}(s-1)} \right\} \breve{{\mathcal {K}}}_{{hf}}, \end{aligned}$$

where the choice of a specific shape of nanoparticles determines the value of the shape factor (*s*). The working fluid in this study is composed of hybrid particles therefore, the extended version of Eq. (19) contains two parameters $s_1$ and $s_2$, which correspond to graphene and magnesia nanoparticles, respectively. The extended version is communicated as

20 $$\begin{aligned} \breve{{\mathcal {K}}}_{{hnf}}=\left\{ \frac{\varUpsilon _{\text {Gra}}(s_1-1)\big (\breve{{\mathcal {K}}}_{\text {Gra}}-\breve{{\mathcal {K}}}_{1}\big )+\breve{{\mathcal {K}}}_{\text {Gra}} +\breve{{\mathcal {K}}}_{1}(s_1-1)}{\varUpsilon _{\text {Gra}}\big (\breve{{\mathcal {K}}}_{1}-\breve{{\mathcal {K}}}_{\text {Gra}} \big )+\breve{{\mathcal {K}}}_{\text {Gra}}+\breve{{\mathcal {K}}}_{1}(s_1-1)} \right\} \breve{{\mathcal {K}}}_{\text {1}}, \end{aligned}$$

where

21 $$\begin{aligned} \breve{{\mathcal {K}}}_{1}=\left\{ \frac{\varUpsilon _{\text {MgO}}(s_2-1) \big (\breve{{\mathcal {K}}}_{\text {MgO}}-\breve{{\mathcal {K}}}_{\text {Eo}}\big )+\breve{{\mathcal {K}}}_{\text {MgO}} +\breve{{\mathcal {K}}}_{\text {Eo}}(s_2-1)}{\varUpsilon _{\text {MgO}}\big (\breve{{\mathcal {K}}}_{\text {Eo}} -\breve{{\mathcal {K}}}_{\text {MgO}} \big )+\breve{{\mathcal {K}}}_{\text {MgO}}+\breve{{\mathcal {K}}}_{\text {Eo}}(s_2-1)} \right\} \breve{{\mathcal {K}}}_{\text {Eo}}. \end{aligned}$$

In this section, the subscript “*hnf*” stands for hybrid nanofluid, “*nf*” denotes nanofluid, and “*hf*” symbolizes host fluid. For computation purposes, the values of $s_1$ and $s_2$ will be chosen from Table 1. The computational values for thermo-physical features are accessible from Table 2.

**Table 1:** Values of *s* for multiple shapes of nanoparticles^30^.

Shapes	*s*
Tetrahedron	4.0613
Blade	8.3
Lamina	16.1576
Brick	3.7
Column	6.3698

**Table 2:** Numerical values for properties of graphene, engine oil, and magnesia^49,50^.

Properties	Nanoparticles		Host fluid
	Graphene	Magnesia	Engine oil
$\breve{\beta }$	0.8 $\times 10^{-5}$	1.26 $\times 10^{-5}$	7 $\times 10^{-5}$
$\breve{{\textsf{C}}}_p$	790	960	2048
$\breve{{\mathcal {K}}}$	5000	48.4	0.1404
$\breve{\rho }$	2200	3580	863

## Exact solutions of a non-dimensional generalized model

In this section, firstly, the developed model will be made dimension-independent to provide the basis for the application of the fractional derivative. This purpose will be achieved by plugging some unit-independent parameters into fundamental equations and connected constraints. Secondly, expressions for thermal and diffusion fluxes composed of generalized Fourier’s and Fick’s laws will be incorporated in the subsequent dimension-independent system to procure a fractional model. Lastly, a comprehensive mathematical analysis will be carried out for the computation of exact solutions. In this analysis, the fractional model and connected constraints will be treated with the Laplace transform (LT). To achieve the first goal, new quantities are presented as follows

22 $$\begin{aligned} q&=\left( \frac{1}{q_0} \right) {\widetilde{q}}, \quad \Theta =\frac{{\widetilde{\Theta }}-\Theta _\infty }{\Theta _w-\Theta _\infty }, \quad \varPsi =\left( {U_0}{\widetilde{\varPsi }} \right) \frac{1}{\breve{\nu }_{\text {Eo}}}, \quad {\mathcal {J}}=\left( \frac{1}{{\mathcal {J}}_0} \right) \widetilde{{\mathcal {J}}}, \nonumber \\ U&=\frac{{\widetilde{U}}}{U_0}, \quad \uptau =\left( U^2_0 {\widetilde{\uptau }} \right) \frac{1}{\breve{\nu }_{\text {Eo}}}, \quad d_1=(d^* U_0)\frac{1}{\breve{\nu }_{\text {Eo}}}, \quad {\mathcal {C}}=\frac{\widetilde{{\mathcal {C}}}-{\mathcal {C}}_\infty }{{\mathcal {C}}_w-{\mathcal {C}}_\infty }, \nonumber \\ q_0&= \left\{ \breve{{\mathcal {K}}}_{\text {Eo}} (\Theta _w-\Theta _\infty )U_0 \right\} \frac{1}{\breve{\nu }_{\text {Eo}}}, \quad {\mathcal {J}}_0= \left\{ \breve{{\mathcal {D}}}_{\text {Eo}} ({\mathcal {C}}_w-{\mathcal {C}}_\infty )U_0 \right\} \frac{1}{\breve{\nu }_{\text {Eo}}}. \end{aligned}$$

The substitution of thermo-physical expressions and the above-mentioned quantities in Eqs. (1)–(5) returns

23 $$\begin{aligned} Z_1 \left( 1+\lambda _1 \frac{\partial }{\partial \uptau }\right) \frac{\partial U(\varPsi ,\uptau )}{\partial \uptau }&=Z_2 \frac{\partial ^2 U(\varPsi ,\uptau )}{\partial \varPsi ^2}+ Z_3 Gr_1 \left( 1+\lambda _1 \frac{\partial }{\partial \uptau }\right) \Theta (\varPsi ,\uptau ) \nonumber \\&\quad + Z_4 Gr_2 \left( 1+\lambda _1 \frac{\partial }{\partial \uptau }\right) {\mathcal {C}}(\varPsi ,\uptau ), \end{aligned}$$

24 $$\begin{aligned} \frac{\partial \Theta (\varPsi ,\uptau )}{\partial \uptau }&=-\left( \frac{1}{{ Z_5 Pr}}\right) \frac{\partial q(\varPsi ,\uptau )}{\partial \varPsi }, \qquad \qquad \qquad \qquad \end{aligned}$$

25 $$\begin{aligned} \frac{\partial {\mathcal {C}}(\varPsi ,\uptau )}{\partial \uptau }&=-\left( \frac{1}{{Sc}}\right) \frac{\partial {\mathcal {J}}(\varPsi ,\uptau )}{\partial \varPsi }, \qquad \qquad \qquad \qquad \end{aligned}$$

26 $$\begin{aligned} q(\varPsi ,\uptau )&=-Z_6 \frac{\partial \Theta (\varPsi ,\uptau )}{\partial \varPsi }, \qquad \qquad \qquad \qquad \quad \end{aligned}$$

27 $$\begin{aligned} {\mathcal {J}}(\varPsi ,\uptau )&=-Z_7 \frac{\partial {\mathcal {C}}(\varPsi ,\uptau )}{\partial \varPsi }. \qquad \qquad \qquad \qquad \quad \end{aligned}$$

The unit-free forms of pertinent conditions are imparted as

28 $$\begin{aligned}&\text {For} \quad {\uptau } = 0: \quad {U}({\varPsi },\uptau )=0, \quad {\Theta }({\varPsi },\uptau )=0, \quad {\mathcal {{C}}}({\varPsi },\uptau )=0, \quad \qquad \quad \end{aligned}$$

29 $$\begin{aligned}&\text {For} \quad {\varPsi } = 0: \quad {U}({\varPsi },{\uptau })=\left\{ \begin{array}{ll} {\uptau } \quad 0 < \uptau \le 1 \\ 1 \qquad {\uptau }> 1 \end{array}\right. , \quad \mathcal {{C}}({\varPsi },{\uptau })=1, \nonumber \\&\quad {\Theta }({\varPsi },{\uptau })-d \frac{\partial {\Theta }({\varPsi },{\uptau })}{\partial {\varPsi }}=h({\uptau }), \quad \end{aligned}$$

30 $$\begin{aligned}&\text {For} \quad {\varPsi } \rightarrow \infty : \quad {U}({\varPsi },{\uptau }) \rightarrow 0, \quad {\Theta }({\varPsi },{\uptau }) \rightarrow 0, \quad \mathcal {{C}}({\varPsi },{\uptau }) \rightarrow 0, \qquad \quad \end{aligned}$$

where Table 3 provides the parameters that appear in Eqs. (23)–(27).

**Table 3:** Quantities emerging in Eqs. (23)–(27).

Symbols	Quantities	Mathematical representation
$\lambda _1$	Maxwell parameter	$\left( {\widetilde{\lambda }} U_0^2 \right) \frac{1}{\breve{\nu }_{{\text {Eo}}}}$
$Gr_1$	Thermal Grashof number	$\frac{g (\Theta _w-\Theta _\infty )}{U_0^3} \left( \breve{\beta }_\Theta \breve{\nu } \right) _{\text {Eo}}$
*Sc*	Schmidt number	$\left( \frac{\breve{\mu }}{\breve{\rho } \breve{D}} \right) _{\text {Eo}}$
*Pr*	Prandtl number	${ \breve{\mu }}_{_{\text {Eo}}} \left( \frac{\breve{{\textsf{C}}}_p}{\breve{{\mathcal {K}}}}\right) _\text {Eo}$
$Gr_2$	Mass Grashof number	$\frac{g ({\mathcal {C}}_w-{\mathcal {C}}_\infty )}{U_0^3} \left( \breve{\beta }_{\mathcal {C}} \breve{\nu } \right) _{\text {Eo}}$
$Z_1$	Quantities relating thermophysical features and volume fraction of nanoparticles	$\big (1-\varUpsilon _{\text {Gra}} \big ) \left[ 1 +\left( \frac{ \breve{\rho }_{\text {MgO}}}{\breve{\rho }_{\text {Eo}}}-1 \right) \varUpsilon _{\text {MgO}} \right] +\varUpsilon _\text {Gra}\big ( \frac{\breve{\rho }_{\text {Gra}}}{\breve{\rho }_{\text {Eo}}} \big )$
$Z_2$		$\frac{\Big (1-\varUpsilon _{\text {MgO}} \Big )^{-\frac{25}{10}}}{ \Big (1-\varUpsilon _{\text {Gra}} \Big )^{\frac{25}{10}}}$
$Z_3$		$\big (1-\varUpsilon _{\text {Gra}} \big ) \left[ 1 +\left\{ \frac{ \big (\breve{\rho }\breve{\beta }_\Theta \big )_{\text {MgO}}}{\big (\breve{\rho }\breve{\beta }_\Theta \big )_{\text {Eo}}}-1 \right\} \varUpsilon _{\text {MgO}} \right] +\varUpsilon _\text {Gra} \frac{\big (\breve{\rho } \breve{\beta }_\Theta \big )_{\text {Gra}}}{\big (\breve{\rho }\breve{\beta }_\Theta \big )_{\text {Eo}}}$
$Z_4$		$\big (1-\varUpsilon _{\text {Gra}} \big ) \left[ 1 +\left\{ \frac{ \big (\breve{\rho }\breve{\beta }_{\mathcal {C}} \big )_{\text {MgO}}}{\big (\breve{\rho }\breve{\beta }_{\mathcal {C}} \big )_{\text {Eo}}}-1 \right\} \varUpsilon _{\text {MgO}} \right] +\varUpsilon _\text {Gra} \frac{\big (\breve{\rho } \breve{\beta }_{\mathcal {C}} \big )_{\text {Gra}}}{\big (\breve{\rho }\breve{\beta }_{\mathcal {C}} \big )_{\text {Eo}}}$
$Z_5$		$\big (1-\varUpsilon _{\text {Gra}} \big ) \left[ 1 +\left\{ \frac{ \big (\breve{\rho } \breve{{\textsf{C}}}_p \big )_{\text {MgO}}}{\big (\breve{\rho }\breve{{\textsf{C}}}_p \big )_{\text {Eo}}}-1 \right\} \varUpsilon _{\text {MgO}} \right] +\varUpsilon _\text {Gra} \frac{\big (\breve{\rho } \breve{{\textsf{C}}}_p \big )_{\text {Gra}}}{\big (\breve{\rho }\breve{{\textsf{C}}}_p \big )_{\text {Eo}}}$
$Z_6$		$\frac{\breve{{\mathcal {K}}}_{hnf}}{\breve{{\mathcal {K}}}_\text {Eo}}$
$Z_7$		$\left( 1-\varUpsilon _\text {Gra}\right) \left( 1-\varUpsilon _\text {MgO}\right)$

Now, this dimension-independent classical model will be shifted into a fractional setting by generalizing the expressions of thermal and mass fluxes using the Prabhakar fractional derivative. These expressions are receptively supplied as

31 $$\begin{aligned} q(\varPsi ,\uptau )&=-Z_6 \left[ {\mathfrak {D}}^{\eta }_{\zeta , \sigma , \varepsilon }\frac{\partial \Theta (\varPsi ,\uptau )}{\partial \varPsi } \right] , \end{aligned}$$

32 $$\begin{aligned} {\mathcal {J}}(\varPsi ,\uptau )&=-Z_7 \left[ {\mathfrak {D}}^{\eta }_{\zeta , \sigma , \varepsilon }\frac{\partial {\mathcal {C}}(\varPsi ,\uptau )}{\partial \varPsi } \right] , \end{aligned}$$

where the Prabhakar fractional operator (${\mathfrak {D}}^{\eta }_{\zeta , \sigma , \varepsilon }$) for an arbitrary function ${\mathcal {G}}(u)$ is given as^34^

33 $$\begin{aligned} {\mathfrak {D}}^{\eta }_{\zeta , \sigma , \varepsilon } \big \{{\mathcal {G}}(u) \big \}&={\textsf{E}}^{-\eta }_{\zeta , r-\sigma , \varepsilon } {\mathcal {G}}^{r}(u)=\texttt{e}^{-\eta }_{\zeta , r-\sigma } (\varepsilon ;u)*{\mathcal {G}}^{r}(u) \nonumber \\&=\int \limits _{0}^{u}(u-\uptau )^{r-\sigma -1}{\textsf{E}}^{-\eta }_{\zeta , r-\sigma }(\varepsilon (u-\uptau )^\zeta ){\mathcal {G}}^{r}(\uptau )d \uptau , \end{aligned}$$

where

34 $$\begin{aligned} {\textsf{E}}^\eta _{\zeta , \sigma , \varepsilon } \left\{ {\mathcal {G}}(u) \right\} =\int \limits _{0}^u (u-\uptau )^{(\sigma -1)}{\textsf{E}}^\eta _{\zeta , \sigma } \big (\varepsilon (u-\uptau )^\zeta \big ) {\mathcal {G}}(\uptau ) d \uptau , \end{aligned}$$

is the Prabhakar integral. The Mittag–Leffler function composed of three parameters and the Prabhakar kernel are given as^51^

$$\begin{aligned} {\textsf{E}}^\eta _{\zeta , \sigma } (p)&= \sum \limits _{j=0}^{\infty } \frac{p^j \Gamma (j+\eta ) }{j! \Gamma (\eta ) \Gamma (\sigma +j \zeta )}, \\ \texttt{e}^\eta _{\zeta , \sigma } (\varepsilon ; u)&=u^{\sigma -1} {\textsf{E}}^\eta _{\zeta , \sigma } \big (\varepsilon u^\zeta \big ). \ \end{aligned}$$

The application of LT on the Prabhakar fractional operator yields the following expression

35 $$\begin{aligned} {\mathfrak {L}}\Big [ {\mathfrak {D}}^\eta _{\zeta , \sigma , \varepsilon } \big \{ {\mathcal {G}}(u) \big \} \Big ]&={\mathfrak {L}} \Big [\texttt{e}^{-\eta }_{\zeta , r-\sigma } (\varepsilon ; u)* {\mathcal {G}}^{(r)}(u) \Big ] \nonumber \\&= {\mathfrak {L}} \Big [\texttt{e}^{-\eta }_{\zeta , r-\sigma } (\varepsilon ; u) \Big ] {\mathfrak {L}} \Big [ {\mathcal {G}}^{(r)}(u) \Big ] \nonumber \\&= \delta ^{\sigma -r}\left( 1-\varepsilon \delta ^{-\zeta } \right) ^\eta \ {\mathfrak {L}} \Big [ {\mathcal {G}}^{r}(u) \Big ]. \end{aligned}$$

### Mathematical analysis of temperature function

The new forms of temperature equation (Eq. (24)), Fourier law (Eq. (31)), and connected constraints (Eqs. (29) and (30)) that derived through the utilization of LT are respectively imparted as

36 $$\begin{aligned} \delta {\overline{\Theta }}(\varPsi , \delta )&=-\frac{1}{Z_5 Pr} \frac{d {\overline{q}}(\varPsi , \delta )}{d \varPsi }, \qquad \qquad \qquad \qquad \end{aligned}$$

37 $$\begin{aligned} \frac{1}{Z_6}{\overline{q}}(\varPsi , \delta )&=-\left\{ \delta ^{\sigma }\left( 1-\varepsilon \delta ^{-\zeta } \right) ^\eta \right\} \frac{d {\overline{\Theta }}(\varPsi , \delta )}{d \varPsi }, \qquad \qquad \qquad \end{aligned}$$

38 $$\begin{aligned} {{\overline{\Theta }}}(0,\delta )-d_1 \frac{d {\overline{\Theta }}(\varPsi ,\delta )}{d \varPsi } \bigg |_{\varPsi =0}&={\overline{h}}(\delta ) \quad \text {and} \quad {\overline{\Theta }}(\varPsi ,\delta ) \rightarrow 0 \quad \text {for} \quad \varPsi \rightarrow \infty , \end{aligned}$$

where $\delta$ is the transformation parameter. Now, taking the derivative of Eq. (37) and plugging the subsequent equation in Eq. (36) yield

39 $$\begin{aligned} \frac{d^2 {\overline{\Theta }}(\varPsi , \delta )}{d \varPsi ^2}=\frac{Z_5 Pr}{Z_6} \left\{ \frac{\delta }{\delta ^{\sigma }\left( 1-\varepsilon \delta ^{-\zeta } \right) ^\eta } \right\} {\overline{\Theta }}(\varPsi , \delta ). \end{aligned}$$

The solution of the above equation is calculated using associated constraints (Eq. (38)), and the simplified form of the temperature function is provided as follows

40 $$\begin{aligned} {\overline{\Theta }}(\varPsi , \delta )=\frac{{\overline{h}}(\delta )}{1+d_1 \sqrt{M(\delta )}} \exp \left( - \varPsi \sqrt{M(\delta )} \right) , \end{aligned}$$

where

$$\begin{aligned} M(\delta )=\frac{\alpha _1 \delta }{\delta ^{\sigma }\left( 1-\varepsilon \delta ^{-\zeta } \right) ^\eta }, \quad \alpha _1=\frac{Z_5 Pr}{Z_6}. \end{aligned}$$

To conveniently utilize the inverse Laplace transform (ILT), Eq. (40) is converted into series form as follows

41 $$\begin{aligned} {\overline{\Theta }}(\varPsi , \delta )&={\overline{h}}(\delta ) \sum \limits _{j=0}^{\infty } (-1)^j \left( d_1\sqrt{M(\delta )} \right) ^j \sum \limits _{k=0}^{\infty } \frac{\left( - \varPsi \sqrt{M(\delta )} \right) ^k}{k!} \nonumber \\&={\overline{h}}(\delta ) \sum \limits _{j=0}^{\infty } \frac{\left( d_1 \sqrt{\alpha _1} \right) ^j}{\delta ^{({\sigma }-1)\frac{j}{2}}\left( 1-\varepsilon \delta ^{-\zeta } \right) ^{\frac{j \eta }{2}}} \sum \limits _{k=0}^{\infty } \frac{\left( - \varPsi \sqrt{\alpha _1} \right) ^k}{k! \delta ^{({\sigma }-1)\frac{k}{2}}\left( 1-\varepsilon \delta ^{-\zeta } \right) ^{\frac{k \eta }{2}}}. \end{aligned}$$

The inverse transformation of the above equation into original coordinates $(\varPsi ,\uptau )$ is procured as

42 $$\begin{aligned} \Theta (\varPsi , \uptau )=h(\uptau )*A_1(\varPsi , \uptau )*A_2(\varPsi , \uptau ), \end{aligned}$$

where

$$\begin{aligned} A_1&=\sum \limits _{j=0}^{\infty } (-d_1)^j (\alpha _1)^{\frac{j}{2}} \uptau ^{(\sigma -1)\frac{j}{2}-1} {\textsf{E}}^{\frac{j \eta }{2}}_{\zeta , (\sigma -1)\frac{j}{2}} \left( \varepsilon \uptau ^\zeta \right) , \\ A_2&=\sum \limits _{k=0}^{\infty } \frac{(-\varPsi )^k}{k!} (\alpha _1)^{\frac{k}{2}} \uptau ^{(\sigma -1)\frac{k}{2}-1} {\textsf{E}}^{\frac{k \eta }{2}}_{\zeta , (\sigma -1)\frac{k}{2}} \left( \varepsilon \uptau ^\zeta \right) . \end{aligned}$$

### Mathematical analysis of diffusion equation

The transformed versions of diffusion equation (Eq. (25)), Fick’s law (Eq. (32)), and respective conditions (Eqs. (29) and (30)) that acquired by the dint of LT are respectively provided as

43 $$\begin{aligned} \delta \overline{{\mathcal {C}}}(\varPsi , \delta )&=-\frac{1}{Sc} \frac{d \overline{{\mathcal {J}}}(\varPsi , \delta )}{d \varPsi }, \qquad \qquad \end{aligned}$$

44 $$\begin{aligned} \frac{1}{Z_7}\overline{{\mathcal {J}}}(\varPsi , \delta )&=-\left\{ \delta ^{\sigma }\left( 1-\varepsilon \delta ^{-\zeta } \right) ^\eta \right\} \frac{d \overline{{\mathcal {C}}}(\varPsi , \delta )}{d \varPsi }, \quad \end{aligned}$$

45 $$\begin{aligned} {\overline{{\mathcal {C}}}}(0,\delta )&=\frac{1}{\delta } \quad \text {and} \quad \overline{{\mathcal {C}}}(\varPsi ,\delta ) \rightarrow 0 \quad \text {for} \quad \varPsi \rightarrow \infty . \end{aligned}$$

Taking the derivative of Eq. (44) and combining the consequent equation with Eq. (43) return

46 $$\begin{aligned} \frac{d^2 \overline{{\mathcal {C}}}(\varPsi , \delta )}{d \varPsi ^2}=\frac{Sc}{Z_7} \left\{ \frac{\delta }{\delta ^{\sigma }\left( 1-\varepsilon \delta ^{-\zeta } \right) ^\eta } \right\} \overline{{\mathcal {C}}}(\varPsi , \delta ). \end{aligned}$$

The Laplace domain concentration function is evaluated considering the relevant conditions (Eq. (45)), and it is supplied as

47 $$\begin{aligned} \overline{{\mathcal {C}}}(\varPsi , \delta )=\frac{1}{\delta } \exp \left( -\varPsi \sqrt{N(\delta )} \right) , \end{aligned}$$

where

$$\begin{aligned} N(\delta )=\frac{\alpha _2 \delta }{\delta ^{\sigma }\left( 1-\varepsilon \delta ^{-\zeta } \right) ^\eta }, \quad \alpha _2=\frac{Sc}{Z_7}. \end{aligned}$$

Equivalently, Eq. (47) is written as

48 $$\begin{aligned} \overline{{\mathcal {C}}}(\varPsi , \delta )&=\frac{1}{\delta } \sum \limits _{m=0}^{\infty } \frac{\left( - \varPsi \sqrt{N(\delta )} \right) ^m}{m!}=\frac{1}{\delta } \sum \limits _{m=0}^{\infty } \frac{\left( - \varPsi \sqrt{\alpha _2} \right) ^m}{m! \delta ^{({\sigma }-1)\frac{m}{2}+1}\left( 1-\varepsilon \delta ^{-\zeta } \right) ^{\frac{\eta m}{2}}}. \end{aligned}$$

Equation (48) is treated with ILT to transmute the concentration function in the real-domain as

49 $$\begin{aligned} {{\mathcal {C}}}(\varPsi , \delta )=\sum \limits _{m=0}^{\infty } \frac{\left( - \varPsi \right) ^m}{m!} (\alpha _2)^{\frac{m}{2}} \uptau ^{({\sigma }-1)\frac{m}{2}} {\textsf{E}}^{\frac{m \eta }{2}}_{\zeta , (\sigma -1)\frac{m}{2}+1} \left( \varepsilon \uptau ^\zeta \right) . \end{aligned}$$

### Mathematical analysis of flow function

After implementation of LT, the flow equation (Eq. (23)) and relevant conditions (Eqs. (29) and (30)) adopt the following form

50 $$\begin{aligned}&\big \{ Z_1 \left( 1+\lambda _1 \delta \right) \delta \big \} {\overline{U}}(\varPsi ,\delta )=Z_2 \frac{\partial ^2 {\overline{U}}(\varPsi ,\delta )}{\partial \varPsi ^2}+ Z_3 Gr_1 \left( 1+\lambda _1 \delta \right) {\overline{\Theta }}(\varPsi ,\delta ) + Z_4 Gr_2 \left( 1+\lambda _1 \delta \right) \overline{{\mathcal {C}}}(\varPsi ,\delta ), \end{aligned}$$

51 $$\begin{aligned}&U(0,\delta )=\frac{1-\exp (-\delta )}{\delta ^2} \quad \text {and} \quad U(\varPsi ,\delta ) \rightarrow 0 \quad \text {for} \quad \varPsi \rightarrow \infty . \qquad \qquad \qquad \end{aligned}$$

Using expressions of ${\overline{\Theta }}(\varPsi ,\delta )$ from Eq. (40) and $\overline{{\mathcal {C}}}(\varPsi ,\delta )$ from Eq. (47) in Eq. (50), and rearranging the subsequent equation yield

52 $$\begin{aligned} Z_2 \frac{\partial ^2 {\overline{U}}(\varPsi ,\delta )}{\partial \varPsi ^2}-\big \{ Z_1 \left( 1+\lambda _1 \delta \right) \delta \big \} {\overline{U}}(\varPsi ,\delta )&= - \left\{ Z_4 Gr_2 \left( 1+\lambda _1 \delta \right) \frac{1}{\delta } \right\} \exp \left( -\varPsi \sqrt{N(\delta )} \right) \nonumber \\&\quad - \Big \{Z_3 Gr_1 \left( 1+\lambda _1 \delta \right) \Big \} \frac{{\overline{h}}(\delta )}{1+d_1 \sqrt{M(\delta )}} \exp \left( - \varPsi \sqrt{M(\delta )} \right) . \end{aligned}$$

The flow function is derived using connected constraints from Eq. (51), and it is provided as

53 $$\begin{aligned} {\overline{U}}(\varPsi ,\delta )&=\frac{1-e^{-\delta }}{\delta ^2} e^{-\varPsi \sqrt{R(\delta )}} +\frac{\alpha _4(1+\lambda _1 \delta ) {\bar{h}}(\delta )}{1+d_1 \sqrt{M(\delta )}} \left[ \frac{e^{-\varPsi \sqrt{R(\delta )}} -e^{ -\varPsi \sqrt{M(\delta )} }}{M(\delta )-\delta (1+\lambda _1 \delta )\alpha _3} \right] \nonumber \\&\quad +\frac{(1+\lambda _1 \delta )\alpha _5}{\delta } \left[ \frac{e^{-\varPsi \sqrt{R(\delta )}}-e^{-\varPsi \sqrt{N(\delta )}}}{N(\delta )-\delta (1+\lambda _1 \delta )\alpha _3} \right] \nonumber \\&= \frac{1-e^{-\delta }}{\delta ^2} e^{-\varPsi \sqrt{R(\delta )}} + \frac{(1+\lambda _1 \delta )\alpha _4 }{\delta (1+\lambda _1 \delta )\alpha _3-M(\delta )} \left[ \frac{{\overline{h}}(\delta )e^{-\varPsi \sqrt{M(\delta )}}}{1+d_1 \sqrt{M(\delta )}}-\frac{{\overline{h}}(\delta )e^{-\varPsi \sqrt{R(\delta )}}}{1+d_1 \sqrt{M(\delta )}} \right] \nonumber \\&\quad + \frac{(1+\lambda _1 \delta )\alpha _5 }{\delta (1+\lambda _1 \delta )\alpha _3-N(\delta )} \left[ \frac{e^{-\varPsi \sqrt{N(\delta )}}}{\delta }-\frac{e^{-\varPsi \sqrt{R(\delta )}}}{\delta } \right] , \end{aligned}$$

where

$$\begin{aligned} \alpha _3=\frac{Z_1}{Z_2}, \quad \alpha _4=\frac{Z_3 Gr_1}{Z_2}, \quad \alpha _5=\frac{Z_4 Gr_2}{Z_2}, \quad R(\delta )=\alpha _3(1+\lambda _1 \delta ) \delta . \end{aligned}$$

The more simplified version of Eq. (53) is communicated as

54 $$\begin{aligned} {\overline{U}}(\varPsi ,\delta )&=\alpha _4 {\overline{U}}_1(\varPsi ,\delta ) \left[ {\overline{\Theta }}(\varPsi ,\delta )-{\overline{h}}(\delta ){\overline{U}}_2(\varPsi ,\delta ) \right] \nonumber \\&\quad +\alpha _5 {\overline{U}}_3(\varPsi ,\delta ) \left[ \overline{{\mathcal {C}}}(\varPsi ,\delta )-{\overline{U}}_4(\varPsi ,\delta ) \right] +\frac{1-e^{-\delta }}{\delta ^2}{\overline{U}}_5(\varPsi ,\delta ), \end{aligned}$$

where

$$\begin{aligned} {\overline{U}}_1(\varPsi ,\delta )&=\frac{(1+\lambda _1 \delta ) }{\delta (1+\lambda _1 \delta )\alpha _3-M(\delta )}, \quad {\overline{U}}_2(\varPsi ,\delta )=\frac{e^{-\varPsi \sqrt{R(\delta )}}}{1+d_1 \sqrt{M(\delta )}}, \\ {\overline{U}}_3(\varPsi ,\delta )&=\frac{(1+\lambda _1 \delta ) }{\delta (1+\lambda _1 \delta )\alpha _3-N(\delta )}, \quad {\overline{U}}_4(\varPsi ,\delta )=\frac{e^{-\varPsi \sqrt{R(\delta )}}}{\delta }, \ \ \\ {\overline{U}}_5(\varPsi ,\delta )&= e^{-p_2\varPsi \sqrt{(\delta +p_1)^2-p_1^2 }}, \quad p_1=\frac{1}{2 \lambda _1}, \quad p_2=\sqrt{\lambda _1 \alpha _3}. \ \ \end{aligned}$$

The final version of the velocity function in primary coordinates $(\varPsi ,\uptau )$ is obtained as

55 $$\begin{aligned} U(\varPsi ,\uptau )&=\alpha _6 U_1(\varPsi ,\uptau )* \left[ \Theta (\varPsi ,\uptau )-h(\uptau )*U_2(\varPsi ,\uptau ) \right] +\alpha _8 U_3(\varPsi ,\uptau )* \left[ {\mathcal {C}}(\varPsi ,\uptau )-U_4(\varPsi ,\uptau ) \right] \nonumber \\&\quad +\uptau *U_5(\varPsi ,\uptau )-{\mathcal {H}}(\uptau _1)\uptau _1*U_5(\varPsi ,\uptau _1), \end{aligned}$$

where

$$\begin{aligned} U_1(\varPsi ,\uptau )&= \sum \limits _{j=0}^{\infty } \sum \limits _{k=0}^{\infty } \frac{(\alpha _7)^j(-\lambda _1)^k \Gamma (j+k)}{k! \Gamma (j)} \uptau ^{j \sigma -k} {\textsf{E}}^{j \eta }_{\zeta ,j \sigma -k+1}(\varepsilon \uptau ^\zeta ), \quad \alpha _6=\frac{\alpha _4}{\alpha _3}, \\ U_2(\varPsi ,\uptau )&=U_{2,a}(\varPsi ,\uptau )*U_{2,b}(\varPsi ,\uptau ), \quad \alpha _7=\frac{\alpha _1}{\alpha _3}, \quad \alpha _8=\frac{\alpha _5}{\alpha _3}, \quad \alpha _9= \frac{\alpha _2}{\alpha _3}, \\ U_{2,a}(\varPsi ,\uptau )&= \sum \limits _{j=0}^{\infty } (-d_1)^j (\alpha _1)^{\frac{j}{2}}\uptau ^{(\sigma -1)\frac{j}{2}-1} {\textsf{E}}^{\eta \frac{j}{2} }_{\zeta ,(\sigma -1)\frac{j}{2} }(\varepsilon \uptau ^\zeta ), \quad \uptau _1=\uptau _1-1, \\ U_{2,b}(\varPsi ,\uptau )&= \sum \limits _{k=0}^{\infty } \sum \limits _{m=0}^{\infty } \frac{(-\varPsi \sqrt{\alpha _3} )^k (\lambda _1)^{\frac{k}{2}-1} \Gamma \left( 1+\frac{k}{2} \right) }{k!m! \Gamma \left( \frac{k}{2}-m+1 \right) }\frac{\uptau ^{m-k-1}}{\Gamma (m-k)}, \\ U_3(\varPsi ,\uptau )&=\sum \limits _{j=0}^{\infty } \sum \limits _{k=0}^{\infty } \frac{(\alpha _9)^j(-\lambda _1)^k \Gamma (j+k)}{k! \Gamma (j)} \uptau ^{j \sigma -k} {\textsf{E}}^{j \eta }_{\zeta ,j \sigma -k+1}(\varepsilon \uptau ^\zeta ), \\ U_4(\varPsi ,\uptau )&= \sum \limits _{m=0}^{\infty } \sum \limits _{k=0}^{\infty } \frac{(-\varPsi \sqrt{\alpha _3} )^m (\lambda _1)^{\frac{m}{2}-k} \Gamma \left( 1+\frac{m}{2} \right) }{k!m! \Gamma \left( \frac{m}{2}-k+1 \right) }\frac{\uptau ^{k-m}}{\Gamma (k-m+1)}, \\ U_5(\varPsi ,\uptau )&=e^{-p_1 \uptau } \delta _1 (\uptau -p_2 \varPsi )+\left\{ \begin{array}{ll} 0 \quad \qquad \qquad \qquad \qquad \qquad \qquad 0 < \uptau \le p_2 \varPsi \\ \frac{p_1 (p_2 \varPsi ) e^{-p_1 \uptau } }{\sqrt{\uptau ^2-p^2_2\varPsi ^2}} I_1 \left[ p_1 \sqrt{\uptau ^2-p^2_2\varPsi ^2} \right] \quad \uptau > p_2 \varPsi \end{array}\right. . \end{aligned}$$

Here, $\delta _1(.)$ is the Dirac delta function, ${\mathcal {H}}(.)$ denotes the Heaviside step function, $I_1(.)$ represents the first kind of modified Bessel functions, and * symbolizes the convolution product.

### Mathematical analysis of pertinent quantities

The modifications produced in Sherwood number, skin friction coefficient, and Nusselt number because of considered nanoparticles, fractional parameters, and shape impacts are analyzed to investigate the significance of these factors for mass transfer rate, shear stress, and heat-transport efficiency of engine oil. The mathematical formulations for Nusselt number, skin friction coefficient, and Sherwood number are imparted as

56 $$\begin{aligned} Nu&=\left\{ \frac{{\widetilde{q}}\big ({\widetilde{\varPsi }},{\widetilde{\uptau }} \big )}{(\Theta _w-\Theta _\infty )U_0} \right\} \frac{\breve{\nu }_{\text {Eo}}}{\breve{{\mathcal {K}}}_{\text {Eo}}}, \quad C_f=\frac{{\widetilde{S}}\big ({\widetilde{\varPsi }},{\widetilde{\uptau }} \big )}{U_0^2\breve{\rho }_\text {Eo}}, \quad Sh=\left\{ \frac{\widetilde{{\mathcal {J}}}\big ({\widetilde{\varPsi }},{\widetilde{\uptau }} \big )}{({\mathcal {C}}_w-{\mathcal {C}}_\infty )U_0} \right\} \frac{\breve{\nu }_{\text {Eo}}}{\breve{{\mathcal {D}}}_{\text {Eo}}}, \end{aligned}$$

where ${\widetilde{q}}\big ({\widetilde{\varPsi }},{\widetilde{\uptau }} \big )$, ${\widetilde{S}}\big ({\widetilde{\varPsi }},{\widetilde{\uptau }} \big )$, and $\widetilde{{\mathcal {J}}}\big ({\widetilde{\varPsi }},{\widetilde{\uptau }} \big )$ have the following expressions

57 $$\begin{aligned} \left( \frac{1}{\breve{{\mathcal {K}}}_{hnf}} \right) {\widetilde{q}}\big ({\widetilde{\varPsi }},{\widetilde{\uptau }} \big )&=-\frac{\partial {\widetilde{\Theta }} \big ({\widetilde{\varPsi }},{\widetilde{\uptau }} \big )}{\partial {\widetilde{\varPsi }}}\bigg |_{{\widetilde{\varPsi }}=0}, \nonumber \\ \left( 1+{\widetilde{\lambda }} \frac{\partial }{\partial {\widetilde{\uptau }}} \right) {\widetilde{S}}\big ({\widetilde{\varPsi }},{\widetilde{\uptau }} \big )&=\breve{\mu }_{hnf} \frac{\partial {\widetilde{U}} \big ({\widetilde{\varPsi }},{\widetilde{\uptau }} \big )}{\partial {\widetilde{\varPsi }}}\bigg |_{{\widetilde{\varPsi }}=0}, \nonumber \\ \left( \frac{1}{\breve{{\mathcal {D}}}_{hnf}} \right) \widetilde{{\mathcal {J}}}\big ({\widetilde{\varPsi }},{\widetilde{\uptau }} \big )&=-\frac{\partial \widetilde{{\mathcal {C}}} \big ({\widetilde{\varPsi }},{\widetilde{\uptau }} \big )}{\partial {\widetilde{\varPsi }}}\bigg |_{{\widetilde{\varPsi }}=0}. \end{aligned}$$

Putting Eq. (57) into Eq. (56) provides the following final versions of Nusselt number, skin friction coefficient, and Sherwood number

58 $$\begin{aligned} Nu=-Z_6 \frac{\partial \Theta \big (\varPsi ,\uptau \big ) }{\partial \varPsi } \bigg |_{\varPsi =0}, \quad \left( 1+\lambda _1 \frac{\partial }{\partial \uptau } \right) C_f=Z_2\frac{\partial U \big (\varPsi ,\uptau \big ) }{\partial \varPsi } \bigg |_{\varPsi =0}, \quad Sh=-Z_7 \frac{\partial {\mathcal {C}} \big (\varPsi ,\uptau \big ) }{\partial \varPsi } \bigg |_{\varPsi =0}. \end{aligned}$$

## Results and discussion

The principal purpose is the assessment of enhancements in the thermal properties of engine oil as a result of the immersion of magnesia and graphene nanoparticles. The Prabhakar fractional operator is exercised as a generalization tool to establish fractional versions of classical equations. Uniform concentration and slip temperature conditions are considered together with the ramped motion of an infinite vertical bounding wall. The ramped velocity function and buoyancy forces (mass and thermal) are the major factors leading to the instigation of the flow. The governing system of equations is composed of flow, concentration, and energy functions, and it is a partially coupled system. Utilizing the Laplace transform, the generalized system is solved, and solutions comprised of Mittag–Leffler functions are derived. This section is organized to provide these solutions in tabular and graphical forms, which were obtained using MATLAB. The graphical illustrations are presented for slip and no-slip cases and for lower and higher time values. A comparison of classical and fractional solutions is also communicated graphically. Additionally, Nusselt number and skin friction coefficient are comprehensively investigated to analyze several phenomena, for instance, the impacts of column, brick, tetrahedron, blade, and lamina shaped particles on thermal efficacy, escalation in heat transfer rate, and the consequence of altering fractional parameters and volume proportions on shear stress.

**Figure 2:**
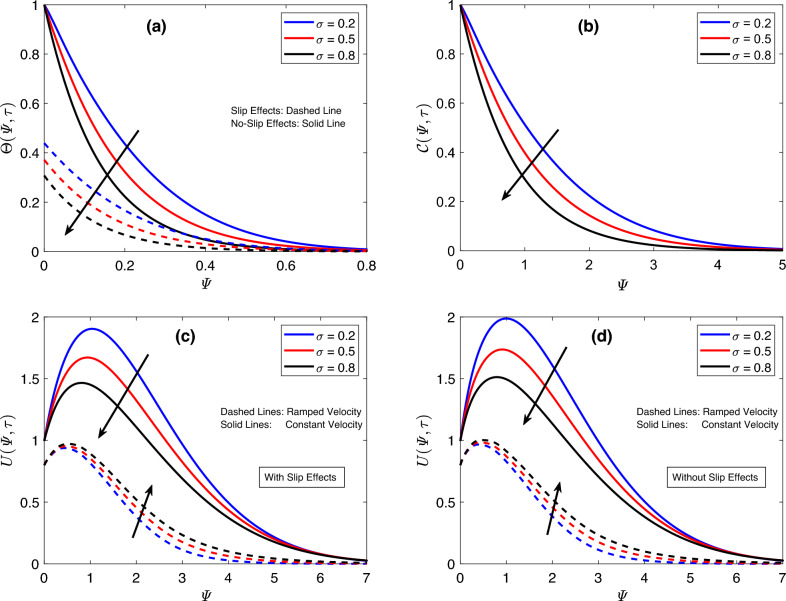
(**a**) Impacts of the parameter $\sigma$ on thermal field. (**b**) Impacts of the parameter $\sigma$ on concentration field. (**c**) Impacts of the parameter $\sigma$ on flow field for slip effects. (**d**) Impacts of the parameter $\sigma$ on flow field for no-slip effects.

**Figure 3:**
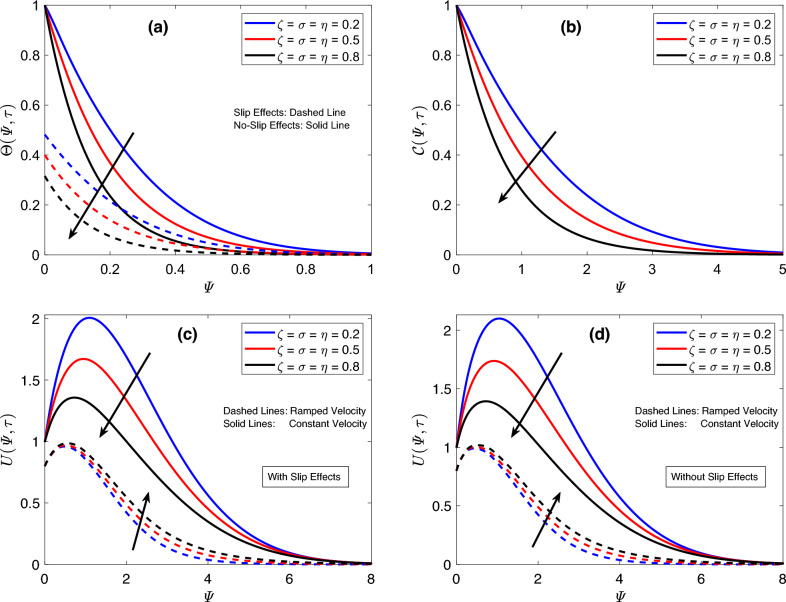
(**a**) Impacts of parameters $\zeta$, $\sigma$, and $\eta$ on thermal field. (**b**) Impacts of parameters $\zeta$, $\sigma$, and $\eta$ on concentration field. (**c**) Impacts of parameters $\zeta$, $\sigma$, and $\eta$ on flow field for slip effects. (**d**) Impacts of parameters $\zeta$, $\sigma$, and $\eta$ on flow field for no-slip effects.

The focus of Fig. 2 is to discuss the implications of altering the fractional parameter $\sigma$. Figure 2a,b indicate a substantial decline in the outcomes of thermal and concentration functions in response to minor enhancements in $\sigma$. For the thermal field, the consequences of modifying $\sigma$ are same whether slip effects are taken into consideration or not. However, when the slip condition is applied, the corresponding temperature graph is always lower than the one associated with the temperature function obtained without the slip condition. The aforesaid finding is likewise valid for velocity graphs. Moreover, an interesting behavior of the flow function is noticed on analyzing the involvement of the parameter $\sigma$. The variations in the flow field for time-dependent and uniform conditions are opposite to each other. The flow profile rises for the ramped case, but subject to the constant condition, the velocity field displays dropping profiles, as demonstrated in Fig. 2c,d. The substantial disturbances in flow, concentration, and heat functions against slight changes in $\sigma$ show that fractional operators are highly efficient for controlling these functions according to the physical situations. Furthermore, the parameter-adjusting property of these operators ensures the acquisition of agreement between theoretical and empirical results. So far, an analysis is made on the basis of a single parameter. However, investigating the behavior of principal functions for modifications in all the involved fractional parameters at the same time is equally essential. For the current study, this task is achieved by plotting the graphs of flow, concentration, and heat functions in Fig. 3 for various values of parameters $\zeta$, $\sigma$, and $\eta$. From Fig. 3a, it is discovered that an increase in the aforementioned parameters reduces the heat function. The response of the concentration field is also the same, as depicted in Fig. 3b. Figure 3c,d disclose that the velocity field behaves in an opposite manner for uniform and time-dependent conditions. It reduces for the earlier case and increases for the later case. The three-parametric kernel, which facilitates velocity profiles to reflect dual patterns, is the main reason for the outcomes discussed earlier. These significant changes in graphs of principle functions for fractional parameters suggest that fractional models present a more thorough explanation of natural phenomena because the details from the earlier step are captured and used in the system in the following step due to the memory properties of the implemented fractional operator. These results indicate that fractional models offer efficient control over boundary layers however, classical models don’t have such features.

**Figure 4:**
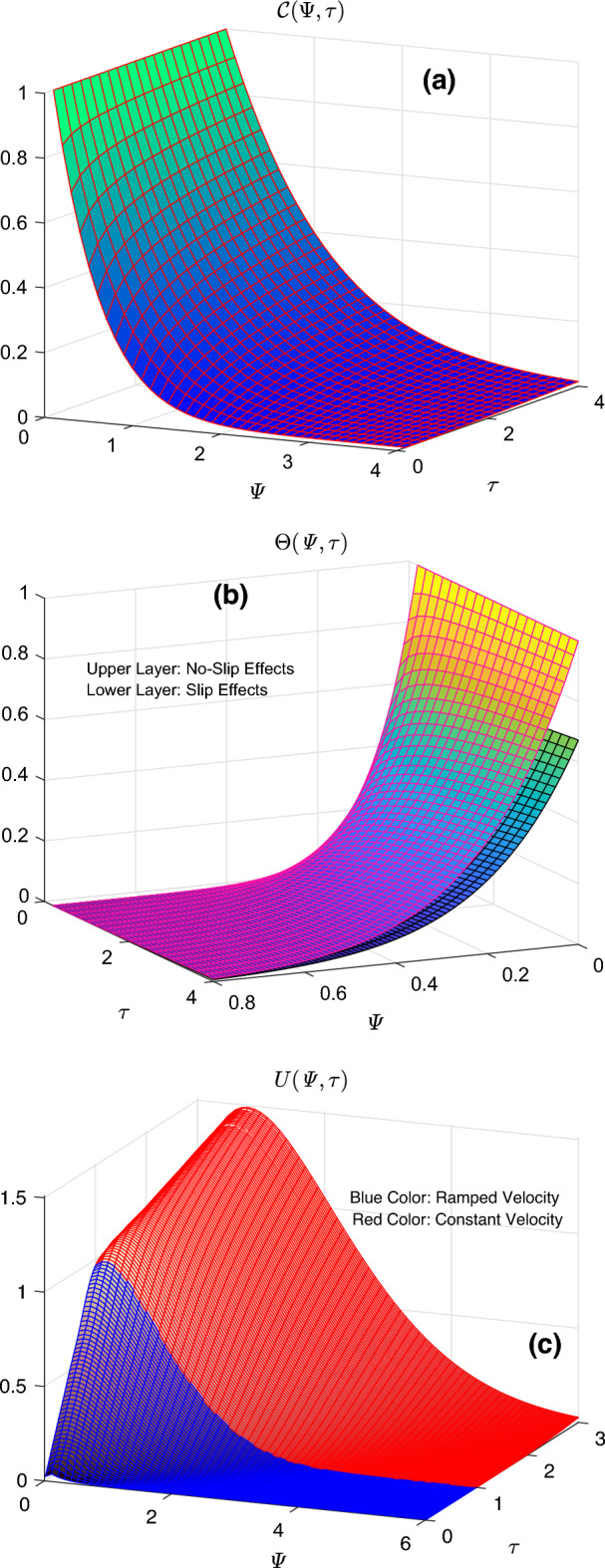
(**a**) Three-dimensional concentration field. (**b**) Three-dimensional thermal field. (**c**) Three-dimensional flow field.

**Figure 5:**
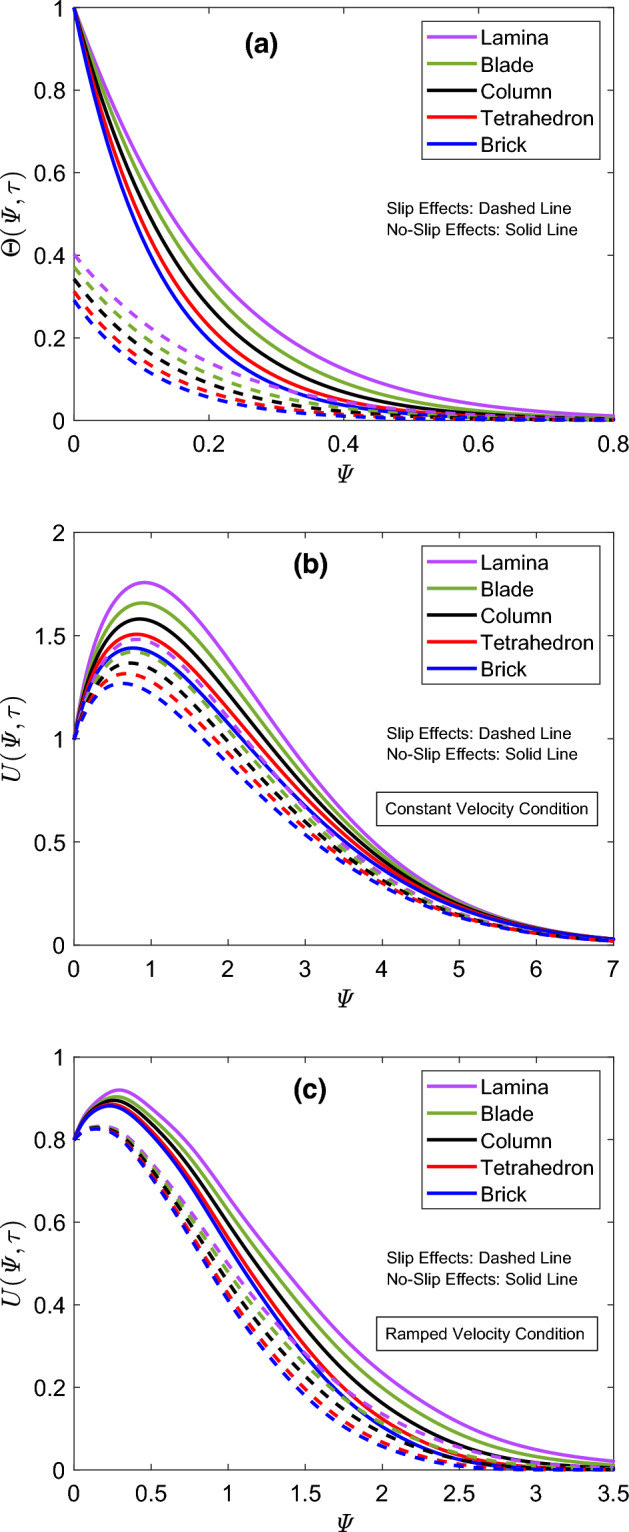
(**a**) Thermal field comparison for multiple shapes. (**b**) Flow field comparison for multiple shapes with constant condition. (**c**) Flow field comparison for multiple shapes with ramped condition.

To thoroughly analyze the significance of applied boundary conditions, three-dimensional demonstrations of concentration, thermal, and flow fields are provided in Fig. 4a–c respectively. It is clear from Fig. 4b that the thermal function has comparatively lower values if slip effects are taken into consideration. Figure 4c contains two regions corresponding to ramped and constant flow cases at the boundary. In the blue region, the flow profile keeps changing the starting point as long as the value of time ($\uptau$) changes. This process continues in the domain $0< \uptau \le 1$. Afterward, the starting point of the flow profile is constant, corresponding to the constant part of the applied flow condition. The respective figure communicates that time variations greatly influence the flow profile for the ramped condition therefore, the use of this condition is helpful for adequately controlling the flow. Figure 5 is constructed to conduct a comparative inspection of flow and thermal profiles for column, lamina, brick, blade, and tetrahedron shapes of nanoparticles. Figure 5a describes that the inclusion of lamina shaped magnesia and graphene nanoparticles in engine oil provides the highest thermal curve. On the other end, the lowest thermal profile is observed when brick shaped particles are considered. The thermal profile exhibits the same pattern for slip and no-slip cases. The shape impacts are included in the mathematical model through the shape factor “*s*”, which depends on the sphericity of nanoparticles. The proportion of the sphere’s surface area to that of real nanoparticles with equal volumes is known as sphericity. The lamina and blade shaped nanoparticles substantially improve the thermal properties of engine oil therefore, the temperature function of the resulted hybrid nanofluid specifies the highest profiles for these shapes. Oppositely, hybrid nanofluid has a relatively weaker thermal conduction ability for brick and tetrahedron shaped nanoparticles. The velocity fields for constant and ramped cases are respectively displayed in Fig. 5b,c. It is perceived that the order of flow profiles for five distinct shapes is identical to that of thermal profiles. In other words, the flow has the maximum velocity for the suspension of lamina shape particles. This profile is respectively followed by blade, column, tetrahedron, and brick shaped particles. These results express that the distribution of tetrahedron and brick shaped particles signifies the viscous effects. On the opposite end, hybrid nanofluid is less viscous when particles have lamina or blade-like shapes. Hence, it flows with greater velocity as depicted in Fig. 5b,c.

**Figure 6:**
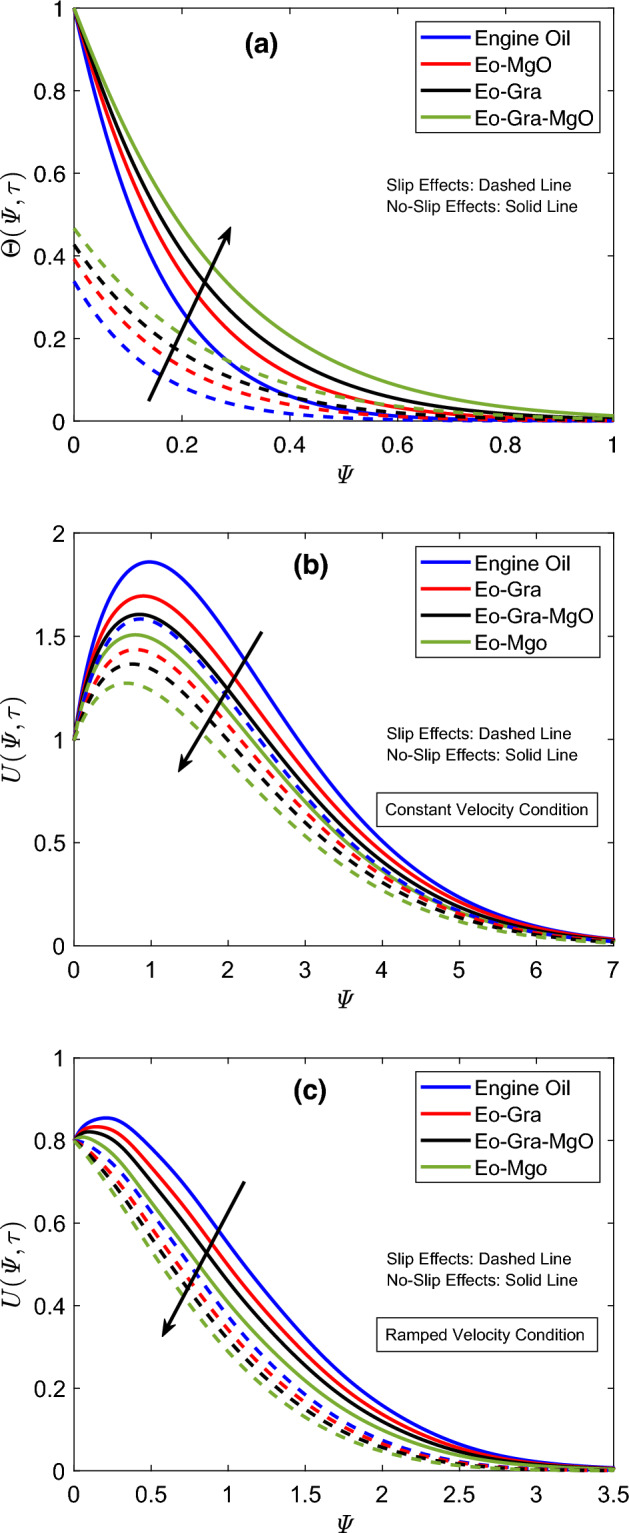
(**a**) Thermal field comparison for different nanofluids. (**b**) Flow field comparison for different nanofluids with constant condition. (**c**) Flow field comparison for different nanofluids with ramped condition.

**Figure 7:**
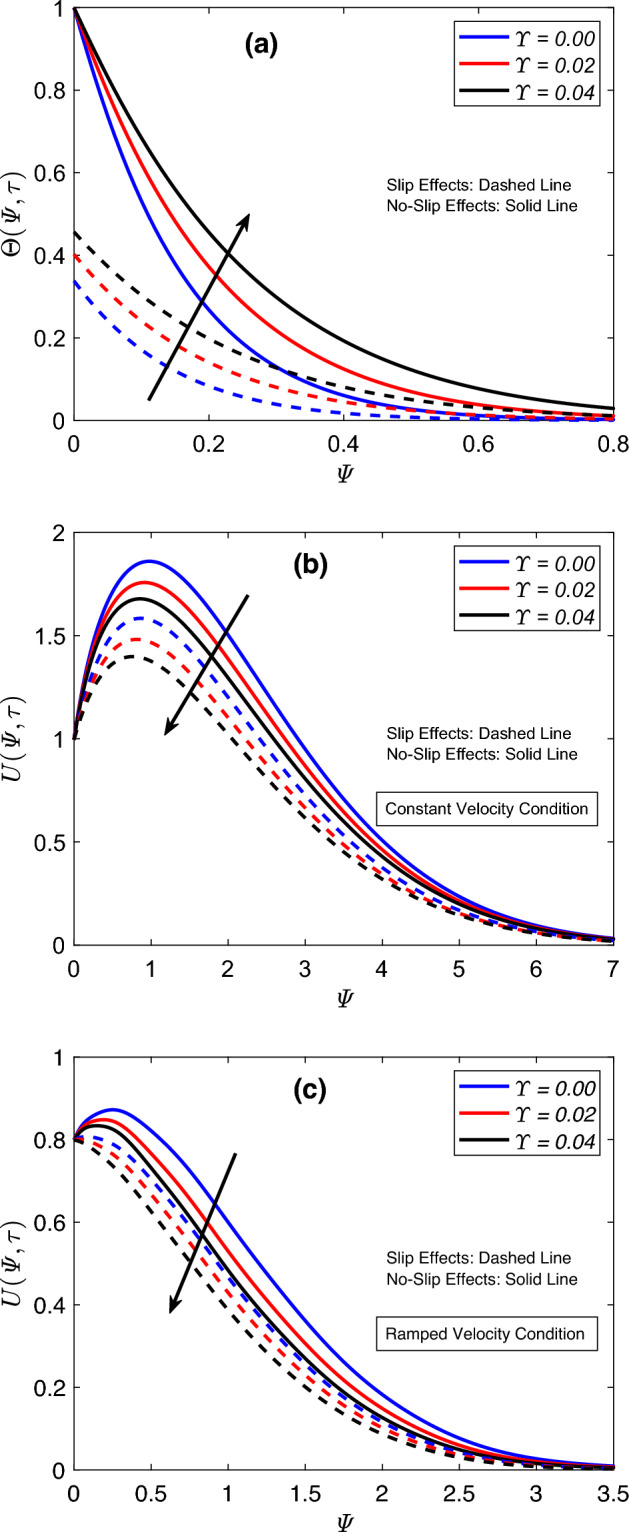
(**a**) Effects of $\varUpsilon$ on thermal field. (**b**) Effects of $\varUpsilon$ on velocity field for constant condition. (**c**) Effects of $\varUpsilon$ on velocity field for ramped condition.

Figure 6 is created to compare temperature and flow distributions for different combinations of engine oil, magnesia, and graphene nanoparticles. In this figure, Eo–MgO is a magnesia based nanofluid, and relevant graphs are obtained by substituting $\varUpsilon _{\text {Gra}}=0$ in mathematical relations. Likewise, graphene based nanofluid is denoted with Eo–Gra. The figures are prepared for this case by placing $\varUpsilon _{\text {MgO}}=0$ in the final solutions. Eo–Gra–MgO is the main hybrid nanofluid of this work, which contains uniform fractions of both nanoparticles. According to Fig. 6a, the temperature distribution of engine oil receives maximum augmentation when both nanoparticles are dispersed in equal proportions. Contrary to this, pure engine oil shows the lowest values for the thermal function, which is somewhat obvious considering its insignificant thermal attributes. In comparison to the thermal conductivity of magnesia, graphene possesses a substantially greater thermal conductivity therefore, the temperature graph of Eo–Gra is relatively higher than that of Eo–MgO. Figure 6b,c reveal that the flow velocity of engine oil is higher than the velocities of other investigated combinations. This highest flow curve is followed by Eo–Gra, Eo–Gra–MgO, and Eo–MgO in the respective order. The conspicuous difference in densities of magnesia, engine oil, and graphene is the primary cause of such velocity patterns. In addition, the immersion of nanoparticles in conventional fluids yields more viscous fluids. Therefore, regular engine oil, due to its lowest density and comparatively weaker viscous nature, indicates the highest flow speed. However, amalgamating engine oil with uniform concentrations of magnesia and graphene considerably disturbs its density. As a result, hybrid nanofluid has a lower density when equated with the density of Eo–MgO. On the contrary, its density is higher than that of graphene based nanofluid. Figure 7 illustrates how the boundary layers of flow and temperature are affected by the rise in total proportion ($\varUpsilon$) of magnesia and graphene particles. Figure 7a depicts that enhancements in the total proportion elevate the temperature graph. This figure further describes that the solution of the heat equation has minimum magnitudes for a zero value of $\varUpsilon$, which indicates that nanoparticles have no physical involvement. These notable differences in thermal field graphs demonstrate that the heat-transportation propensity of pure engine oil is highly ineffective for industrial processes. However, when engine oil is hybridized with magnesia and graphene nanoparticles, its heat-carrying capacity is boosted because of the strong intrinsic features of suspended nanoparticles, which improves the functionality of the emerging hybrid nanofluid. The ensuing hybrid nanofluid, because of the boost in thermal features, absorbs heat comparatively faster and in a greater amount; therefore, temperature fluctuations at the boundary wall occur expeditiously. Consequently, Fig. 7a depicts higher thermal profiles that signify a considerable temperature rise. As far as the flow distribution is concerned, the inverse conduct of the velocity profile is discerned from Fig. 7b,c. The corresponding figures further communicate that the immersion of magnesia and graphene nanoparticles leads to producing a pronounced drop in the flow velocity. The viscosity of the host fluid keeps increasing as long as the concentration of nanoparticles continues to rise, which is one of the pivotal characteristics of nanoparticles in the physical sense. This viscosity augmentation results in reduced flow speed therefore, a decline in velocity profile is seen in the respective figures. In addition, temperature slip effects also decelerate the flow.

**Figure 8:**
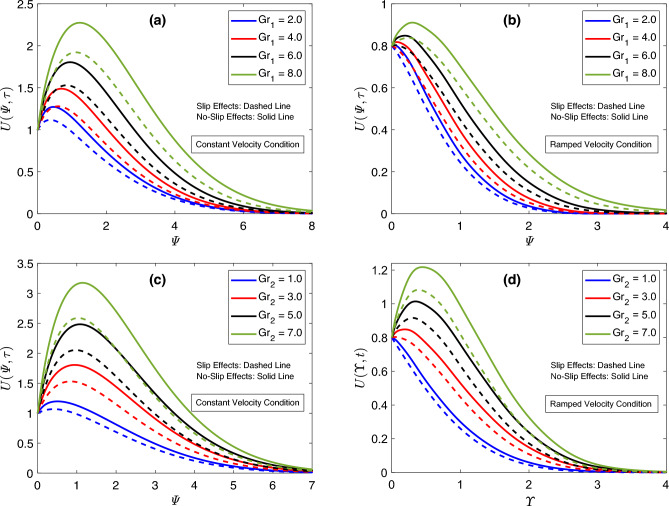
(**a**) Impacts of $Gr_1$ on flow field for constant condition. (**b**) Impacts of $Gr_1$ on flow field for ramped condition. (**c**) Impacts of $Gr_2$ on flow field for constant condition. (**d**) Impacts of $Gr_2$ on flow field for ramped condition.

**Figure 9:**
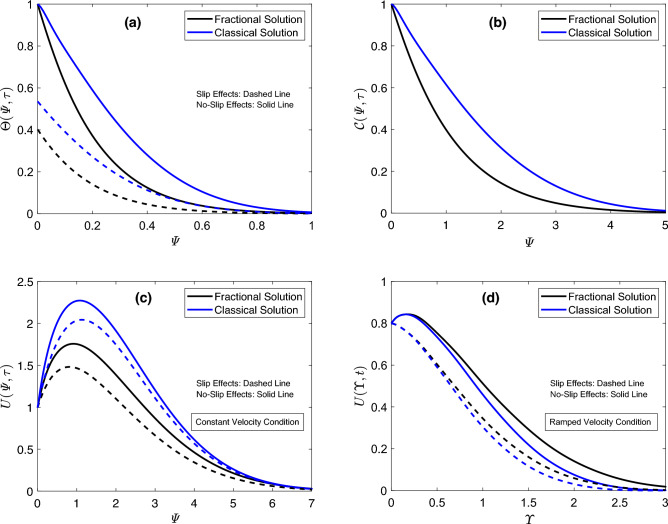
(**a**) Thermal field comparison. (**b**) Concentration field comparison. (**c**) Flow field comparison with constant condition. (**d**) Flow field comparison with ramped condition.

The response of the flow distribution under the relative dominant and weak actions of several forces, like viscous, and thermal and diffusive buoyancy forces, is reported in Fig. 8. Due to the varied characteristics of these forces, flow development is either aided or resisted. In this work, $Gr_1$ symbolizes the thermal Grashof number, which is influenced by the temperature gradient. In the mathematical sense, the thermal buoyancy force indicates a direct association with $Gr_1$, whereas viscous forces and $Gr_1$ share an inverse correspondence. Similarly, the mass Grashof number is characterized by $Gr_2$, which shows a direct connection with the diffusive buoyancy force and is associated with viscous forces in an opposite manner. Figure 8a,b exhibit that positive alterations in $Gr_1$ heighten the flow graphs. An identical result for flow patterns against the modification of $Gr_2$ can be perceived from Fig. 8c,d for constant and ramped velocity cases, respectively. Physically, the elevating values of $Gr_1$ and $Gr_2$ specify that the boundary wall has a comparatively enhanced temperature and concentration gradient is strong. The conventional currents eventually emerge as a consequence of concentration changes and additional heating disturbing the density. Ultimately, the viscous force is left with a negligible contribution since convectional currents not only yield the buoyant force but also assist in augmenting its intensity. Thus, the deformation faces no significant resistance; therefore, the hybrid nanofluid indicates a greater velocity, and a rise in the corresponding curve can be followed from Fig. 8. A comparative study of concentration, velocity, and heat equations for regular and fractional models is conducted by the dint of Fig. 9. Figure 9a,b describe that thermal and concentration solutions derived using the fractional approach have minimum outputs than those established employing the conventional model. Furthermore, there is no influence of the slip condition on the temperature field in this regard. However, the graphical outcomes of the velocity distribution are very interesting because the respective condition also has a significant contribution in this case. For the uniform condition, the behavior of the velocity field is identical to the aforementioned behaviors of thermal and concentration functions. In this case, the fractional-order solution demonstrates a profile lower than that representing the solution procured via the classical model. Whereas, the ordering of velocity profiles changes when a ramped condition is considered. In this case, the graph of the velocity solution having fractional order is higher. Furthermore, it is discerned that applying the temperature slip condition provides lower graphs of energy and flow distributions as compared to the graphs prepared in the absence of this condition. The results in Fig. 9 also endorse the fact that fractional models, because of their memory features and order-adjustment characteristics, are more effective for an extensive and accurate description of physical mechanisms.

**Figure 10:**
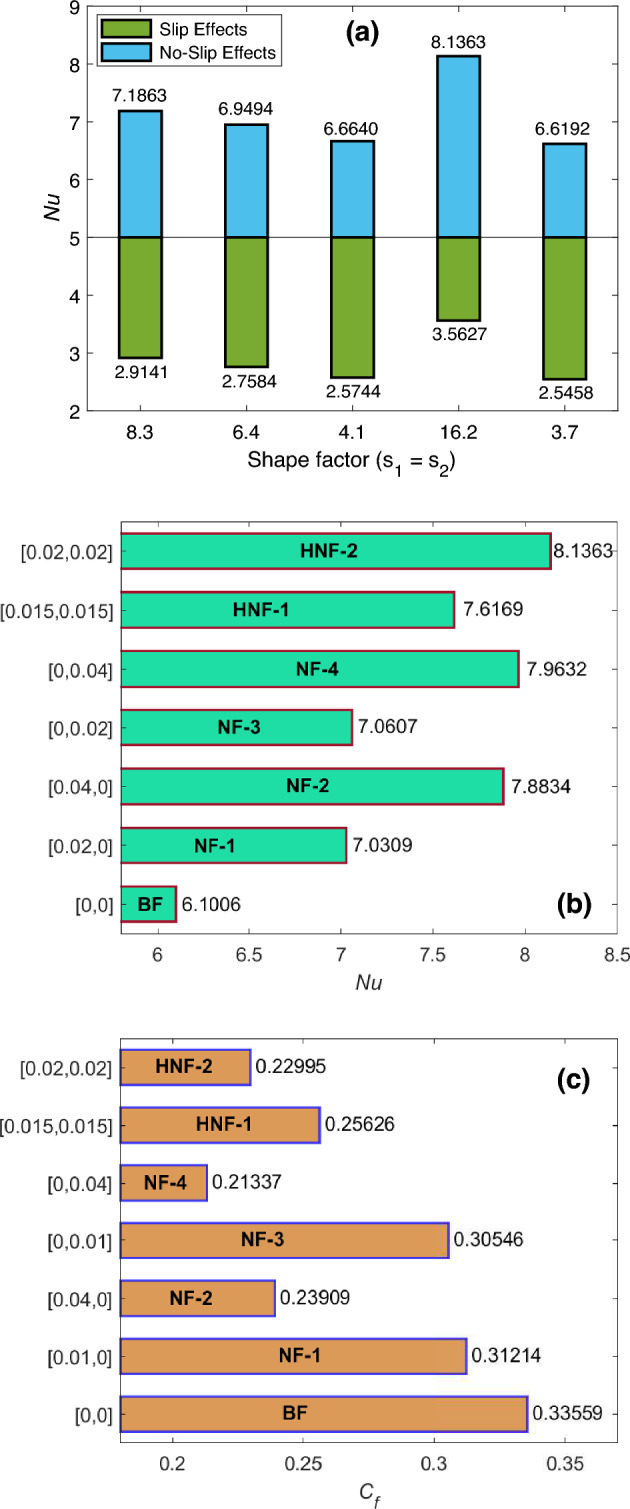
(**a**) *Nu* comparison for multiple shapes. (**b**) *Nu* comparison for different nanofluids. (**c**) $C_f$ comparison for different nanofluids.

A comparative evaluation of Nusselt number (*Nu*) for slip and no-slip effects and also for multiple shapes of working hybrid nanoparticles is carried out with the aid of Fig. 10a. It is spotted that improvement in *Nu* is not the same for each type of considered shape. For instance, the immersion of lamina shaped magnesia and graphene nanoparticles yields the maximum increment in the value of *Nu*. Contrary to that, the minimum boost in heat transfer rate is witnessed when particles have brick shapes. On the basis of this observation, it is concluded that the most effective shape of nanoparticles to ameliorate the thermal efficacy of industrial fluids is the lamina shape. Furthermore, the consideration of slip effects lowers the outcomes of *Nu*. Figure 10b,c are provided to investigate *Nu* and skin friction coefficient ($C_f$) for multiple single and dual combinations of magnesia and graphene nanoparticles with engine oil. A comparative inspection reveals that *Nu* and $C_f$ behave differently when the particle’s allocation is maximized. Precisely speaking, raising the inputs of $\varUpsilon _{\text {Gra}}$ and $\varUpsilon _{\text {MgO}}$ from 0.00 to 0.02 results in the escalation of *Nu* and diminution of $C_f$. In Fig. 10b,c, the specific proportion of nanoparticles for each presented combination is mentioned along the $y-$axis. Figure 10b displays that when magnesia and graphene particles have maximal and identical fractions ($\varUpsilon _{\text {Gra}}=0.02=\varUpsilon _{\text {MgO}}$), *Nu* produces the highest outcome in comparison to other examined amalgamations. Figure 10c depicts that the smallest output of $C_f$ is for nanofluid having magnesia nanoparticles. Table 4 is organized to chew over the implications of changing the volume concentrations of hybrid particles for the thermal usability of engine oil. Moreover, the amelioration in heat transport rate is anticipated in terms of percentage. It is found that a slight enhancement in $\varUpsilon$ induces a pronounced rise in *Nu*. Table 4 indicates a boost of 33.37% in thermal effectiveness when magnesia and graphene nanoparticles attain maximum fractions ($\varUpsilon _{\text {Gra}}=0.02$ and $\varUpsilon _{\text {MgO}}=0.02$) during hybridization. This improvement in *Nu* is quite substantial and supports the employment of hybrid nanofluid that is being analyzed in processes where one of the essential focuses is the efficient cooling of conduits.

To fully understand the involvement of each considered shape of nanoparticles in strengthening the thermal potential of carrier fluid, Table 5 is developed for seven different values of $\varUpsilon$. These results indicate that lamina-shaped particles are the most significant when it comes to uplifting the thermal features because *Nu* is the maximum ($Nu = 8.1363$) for this shape. In this regard, the performance of blade-shaped particles is comparatively weaker than that of lamina-shaped particles. It is noticed that *Nu* for brick-like shapes of particles has the lowest outputs as equated to *Nu* outputs corresponding to other shapes. On making a percentage-based comparison, it is found that the heat transfer rate is only improved up to 8.50% when particles are of brick shapes. These improvements for blade, column, and tetrahedron shapes are 17.79%, 13.91%, and 9.24% in a respective sequence. These noteworthy percentage differences highlight the momentous influences of particles’ shapes on maximizing the industrial functionality of ordinary fluids. These findings emphasize that the shapes of embedded hybrid particles are essential factors for the optimization of insufficient thermal characteristics of traditional fluids. Based on the provided results, it can be concluded that the information extracted through theoretic analyses without accounting for shape impacts may not be fully reliable for practical uses. Table 6 is prepared to examine the influence of parameters $\zeta$, $\sigma$, and $\eta$ on *Nu* for both slip and no-slip cases. It is identified that *Nu* increases due to the enhancement of these parameters. The outcome is the same whether slip effects are taken into consideration or not. In Table 7, the computational results for Sherwood number (*Sh*) are compared for two dissimilar inputs of Schmidt number (*Sc*), taking into consideration the impact of fractional parameters. The table reports that *Sh* gets escalated when the magnitude of *Sc* is high. In this case, the diffusion coefficient is small and the viscous impacts are dominant therefore, a boost in the mass transfer rate occurs. Moreover, the parameters $\zeta$, $\sigma$, and $\eta$ tend to enhance the value of *Sh*. The changes in $C_f$ because of these parameters are investigated by the dint of Table 8. This inspection is conducted for ramped and constant boundary velocity functions. The table describes that $C_f$ follows inverse patterns for two considered cases against the escalation of $\zeta$. For the constant case, $C_f$ gets attenuated, whereas $C_f$ produces rising values for the ramped case. This pattern is also followed, subject to increasing alterations of $\sigma$. As far as the contribution of the parameter $\eta$ is concerned, $C_f$ communicates declining values for the ramped case as well as the constant case. These enhancements and diminutions of *Nu*, *Sh*, and $C_f$ in Tables 6, 7, and 8 are purely dependent on the kernel of the applied fractional operator. The results communicated in these tables accentuate the fact that the fractional model established in this investigation offers more effective control over heat transfer and flow processes as contrasted to regular models. The specificity and correctness of outcomes can be assured via adapting such models by making the necessary modifications to fractional parameters. The considered problem, which involves heat transfer and flow over a vertical surface, has multiple real-life applications, and the presented findings are useful in this regard. For instance, flow over the vertical surfaces of buildings generates wind loads that can affect the structural design and safety of the building. Regarding heat exchangers, flow over the vertical surface is important for heat transfer between fluids. The flow can enhance the heat transfer rate by promoting mixing between fluids and increasing the surface area available for heat transfer. The flow over the vertical surfaces of wind turbine blades has a substantial contribution to generating lift and producing power. Similarly, the flow over vertical cliffs and shorelines can cause coastal erosion by carrying away sediment and rock. Moreover, flow over a vertical surface is commonly used for cooling electronic devices like computer chips, CPUs, and other electronic circuits. These are a few of the numerous applications of the considered problem. The presented outcomes improve the understanding of flow and heat transfer over a vertical surface. With a better understanding of these phenomena, architects and engineers can design better buildings that are more energy-efficient and comfortable for occupants. This will also reduce energy consumption. These results also help the engineers in the development of such cooling systems that remove heat from the surface more effectively. With a better understanding of heat transfer and flow over a vertical surface that this study offers, the fuel efficiency of ships and automobiles can be enhanced. Moreover, these results suggest that engine oil hybridized with magnesia and graphene particles is a more useful fluid for the lubrication of machines as compared to regular fluids. This is also one of the practical applications of our results. Since our results consist of exact solutions, they can be used to verify the numerical techniques formulated for solving the fractional-order models. Our results also enable the possibility of getting a suitable agreement between theoretical outcomes and the experimental data by using the order-variation property of the involved fractional operator.

**Table 4:** Enhancement percentage of *Nu* due to modifications of volume proportions.

$\varUpsilon$	$\zeta$	$\sigma$	$\eta$	*Nu*	Enhancement $\%$
0.00	0.1	0.5	0.8	6.1006	–
0.005	0.1	0.5	0.8	6.3491	4.07
0.01	0.1	0.5	0.8	6.5992	8.17
0.015	0.1	0.5	0.8	6.8510	12.30
0.020	0.1	0.5	0.8	7.1046	16.46
0.025	0.1	0.5	0.8	7.3599	20.64
0.03	0.1	0.5	0.8	7.6169	24.85
0.035	0.1	0.5	0.8	7.8757	29.09
0.040	0.1	0.5	0.8	8.1363	33.37

**Table 5:** Improvement in *Nu* due to various shapes of graphene and magnesia particles.

$\varUpsilon$	*Nu*				
	Tetrahedron	Blade	Column	Lamina	Brick
0.010	6.2388	6.3666	6.3086	6.5992	6.2278
0.015	6.3085	6.5009	6.4136	6.8510	6.2920
0.020	6.3787	6.6361	6.5193	7.1046	6.3566
0.025	6.4494	6.7723	6.6258	7.3599	6.4216
0.030	6.5205	6.9094	6.7329	7.6169	6.4871
0.035	6.5920	7.0474	6.8408	7.8757	6.5529
0.040	6.6640	7.1863	6.9494	8.1363	6.6192

**Table 6:** The influence of parameters $\zeta$, $\sigma$, and $\eta$ on *Nu*.

$\zeta$	$\sigma$	$\eta$	*Nu*	
			No-slip case	Slip case
0.2	0.4	0.9	6.5992	3.4860
0.4	–	–	6.8950	3.5661
0.6	–	–	7.2530	3.6568
0.8	–	–	7.6705	3.7547
0.1	0.1	–	2.6941	3.0410
–	0.3	–	5.2546	3.3171
–	0.6	–	6.2899	3.7104
–	0.9	–	8.0625	4.0822
–	0.4	0.2	6.2515	3.3113
–	–	0.5	6.5565	3.3714
–	–	0.7	6.7674	3.4113
–	–	0.9	6.9845	3.4508

**Table 7:** The influence of parameters $\zeta$, $\sigma$, and $\eta$ on *Sh*.

$\zeta$	$\sigma$	$\eta$	*Sh*	
			$Sc=0.6$	$Sc=2.0$
0.2	0.4	0.9	0.8471	1.5466
0.4	–	–	1.2915	2.3579
0.6	–	–	1.7364	3.1703
0.8	–	–	2.1153	3.8620
0.1	0.1	–	0.3526	0.6437
–	0.3	–	0.5624	1.0267
–	0.6	–	0.8718	1.5918
–	0.9	–	1.2060	2.2019
–	0.4	0.2	0.4005	0.7312
–	–	0.5	0.4993	0.9115
–	–	0.7	0.5766	1.0527
–	–	0.9	0.6646	1.2133

**Table 8:** The influence of parameters $\zeta$, $\sigma$, and $\eta$ on $C_f$.

$\zeta$	$\sigma$	$\eta$	$C_f$	
			Ramped condition	Constant condition
0.2	0.4	0.9	0.1746	2.1965
0.4	–	–	0.1838	2.1166
0.6	–	–	0.1936	2.0313
0.8	–	–	0.2031	1.9438
0.1	0.1	–	0.2553	2.6835
–	0.3	–	0.2869	2.3808
–	0.6	–	0.3272	1.9463
–	0.9	–	0.3592	1.5357
–	0.4	0.2	0.3789	2.4275
–	–	0.5	0.3453	2.3430
–	–	0.7	0.3232	2.2877
–	–	0.9	0.3013	2.2333

## Conclusion

The primary objective of this work is to forecast the escalation in engine oil’s thermal capacity because of its hybridization with magnesia and graphene nanoparticles. To thoroughly inspect the involvement of nanoparticles in disturbing the flow patterns and ameliorating the thermal and material characteristics, nanoparticles are assumed to have column, brick, tetrahedron, blade, and lamina shapes. To conduct this analysis, a fractional model is established by the dint of generalized Fourier’s and Fick’s laws. Ramped flow and temperature slip conditions are jointly taken into account for the first time. The basic governing system is composed of flow, concentration, and energy equations. The Prabhakar fractional operator is implemented to include a multi-parametric kernel into thermal and diffusion flux equations, changing the classical system into a fractional one. The inclusion of dimension-free quantities into basic equations and the application of the Laplace transform are two principal steps for the procurement of solutions. The variations in boundary layers and fall and elevation in profiles of velocity and thermal functions are explicated via graphs. A comparative report on the performance of nanoparticles for several shapes is provided. Moreover, the augmentation in Nusselt number in terms of percentage, shape features, and consequences of modifying the fractional parameters are also investigated. Some comparative illustrations of primary functions, extracted from standard and fractional models, are produced to emphasize the critical impact of fractional techniques for modeling purposes. A brief inspection of shear stress is conducted for fractional parameters and several combinations of graphene, magnesia, and engine oil. The key observations of this analysis are summarized as

- Engine oil’s hybridization with equal proportions of magnesia and graphene nanoparticles provides a 33% amelioration in its thermal efficiency.
- When hybrid particles are evenly immersed, their material properties and shape effects escalate the viscosity, due to which the boiling point of hybrid nanofluid rises. Consequently, its potential for heat transportation enhances, and it possesses higher thermal stability.
- An increment in collective volume fraction leads to raising the profile of the thermal field. However, the flow profile indicates an inverse trend.
- A significant variation in skin friction coefficient for small modifications of fractional parameters demonstrates that the fractional model can adequately control the shear stress.
- The lamina shaped hybrid nanoparticles provide the highest values of Nusselt number.
- When fractional parameters are varied, the flow profile demonstrates inverse patterns for constant and ramped cases.
- Graphene nanoparticles have a markedly greater influence on strengthening the thermal features than magnesia nanoparticles do.
- The buoyancy forces substantially accelerate the flow of hybrid nanofluid.
- In contrast to the slip condition, the thermal curve is higher for the no-slip temperature condition.
- Due to memory features, generalized Fick’s and Fourier’s laws describe diffusion and thermal fluxes more effectively.
- Nusselt number reveals that the highest heat transfer rate is specified by the presented hybrid nanofluid as equated to that of other observed nanofluids and pure engine oil.
- The paired employment of the fractional model and ramped velocity function offers improved flow control.

## Future research recommendations

- The basic model of this study can be modified to investigate flow problems for other geometries such as disks, cylinders, channels, and pipes.
- This model can be extended for two and three-dimensional problems.
- Some new results can be obtained by operating other fractional derivatives for the same problem, and comparative analyses can be conducted.
- The combinations of other rate-type fluids with different nanoparticles can be studied using the appropriately modified version of this model.

## Data Availability

All data generated or analysed during this study are included in this published article.

## List of symbols

$${\widetilde{\lambda }}$$: Maxwell parameter
$${\widetilde{q}}$$: Thermal flux
$${\widetilde{\Theta }}$$: Temperature function
$Gr_1$: Thermal Grashof number
$$\breve{{\mathcal {K}}}$$: Thermal conductivity
$${\widetilde{\varPsi }}$$: Space component
${\mathcal {C}}_\infty$: Free stream concentration
$$U_0$$: Characteristic velocity
$$\widetilde{{\mathcal {C}}}$$: Concentration function
$$\widetilde{{\mathcal {J}}}$$: Diffusion flux
$$\uptau _0$$: Characteristic time
$$\breve{D}$$: Diffusion coefficient
$\varUpsilon$: Proportion of particles
$$\widehat{{\textsf{K}}}$$: Thermal conductivity
*Nu*: Nusselt number
$\breve{{\textsf{C}}}_p$: Specific heat capacity
Eo: Engine oil
Gra: Graphene
BF: Base fluid
$${\widetilde{U}}$$: Velocity function
$$\breve{\rho }$$: Density
$${\widetilde{\uptau }}$$: Time
*g*: Gravitational acceleration
$\breve{\beta }_{\Theta }$: Thermal expansion coefficient
$Gr_2$: Mass Grashof number
$$d^*$$: Slip parameter
$$\breve{\mu }$$: Viscosity
$$\Theta _\infty$$: Ambient temperature
*s*: Shape factor
$\breve{\beta }_{{\mathcal {C}}}$: Mass expansion coefficient
*Sc*: Schmidt number
*Pr*: Prandtl number
$C_f$: Skin friction coefficient
$$\delta$$: Laplace parameter
$$\zeta , \sigma , \eta$$: Fractional parameters
NP: Nanoparticles
MgO: Magnesia
NF-1, NF-2: Graphene based nanofluids
NF-3, NF-4: Magnesia based nanofluids
HNF-1, HNF-2: Hybrid nanofluids
